# Heterogeneity in endpoints, monitoring methods and blanking periods in clinical trials of atrial fibrillation ablation: a systematic review and meta-analysis

**DOI:** 10.1007/s10840-026-02388-6

**Published:** 2026-06-27

**Authors:** Francis J. Ha, Duron Prinsloo, Filip Pesevski, Hui Chen Han, Adam Brown, Nitesh Nerlekar, Emily Kotschet

**Affiliations:** Victorian Heart Institute and Victorian Heart Hospital, Monash University, Clayton, VIC 3168 Australia

**Keywords:** Atrial fibrillation, Catheter ablation, Randomized controlled trials, Blanking period, Cardiac monitoring

## Abstract

**Background:**

Catheter ablation is a cornerstone of rhythm control in atrial fibrillation (AF). It is important that clinical trials in AF ablation have comparable endpoints and outcomes. We performed a systematic review of randomised controlled trials (RCTs) in AF ablation to evaluate endpoint definitions, monitoring methods, follow-up protocols and blanking periods.

**Methods:**

Electronic databases were searched for RCTs in catheter ablation for AF with ≥ 100 patients and ≥ 6 months of follow-up. Data were collected on study characteristics, patient demographics, arrhythmia monitoring methods, blanking periods, arrhythmia endpoints.

**Results:**

129 RCTs (*n* = 31,406) met inclusion criteria; 114 studies involved radiofrequency ablation and 43% patients had persistent AF. There were 19 different definitions of arrhythmia endpoints; the most common was AF/atrial tachyarrhythmia ≥ 30 s (102/129; 79%). Composite endpoints were used in 55 studies (43%). There were 11 different methods of monitoring arrhythmia recurrence; 24-h Holter (67/129; 52%) was most frequent, followed by transtelephonic/event-triggered monitoring (50/129; 39%), then 7-day Holter monitors (30/129; 23%). In the first year of follow-up, arrhythmia recurrence was most commonly assessed at 6 months (120/129; 93%), 12 months (117/129; 91%), and 3 months (116/129; 90%); 56 trials (43%) reported 1-month visit. Most trials used a 90-day blanking period (109/129, 84%). Recurrences during blanking period was reported in only 32 trials (25%).

**Conclusion:**

Significant heterogeneity exists in RCTs of AF ablation with varying definitions of arrhythmia recurrence, monitoring methods, follow-up schedules and blanking periods. Scientific consensus and more standardised assessment of arrhythmia recurrence is needed to improve comparability of clinical outcomes in RCTs of AF ablation.

**Graphical abstract:**

**Figure:**
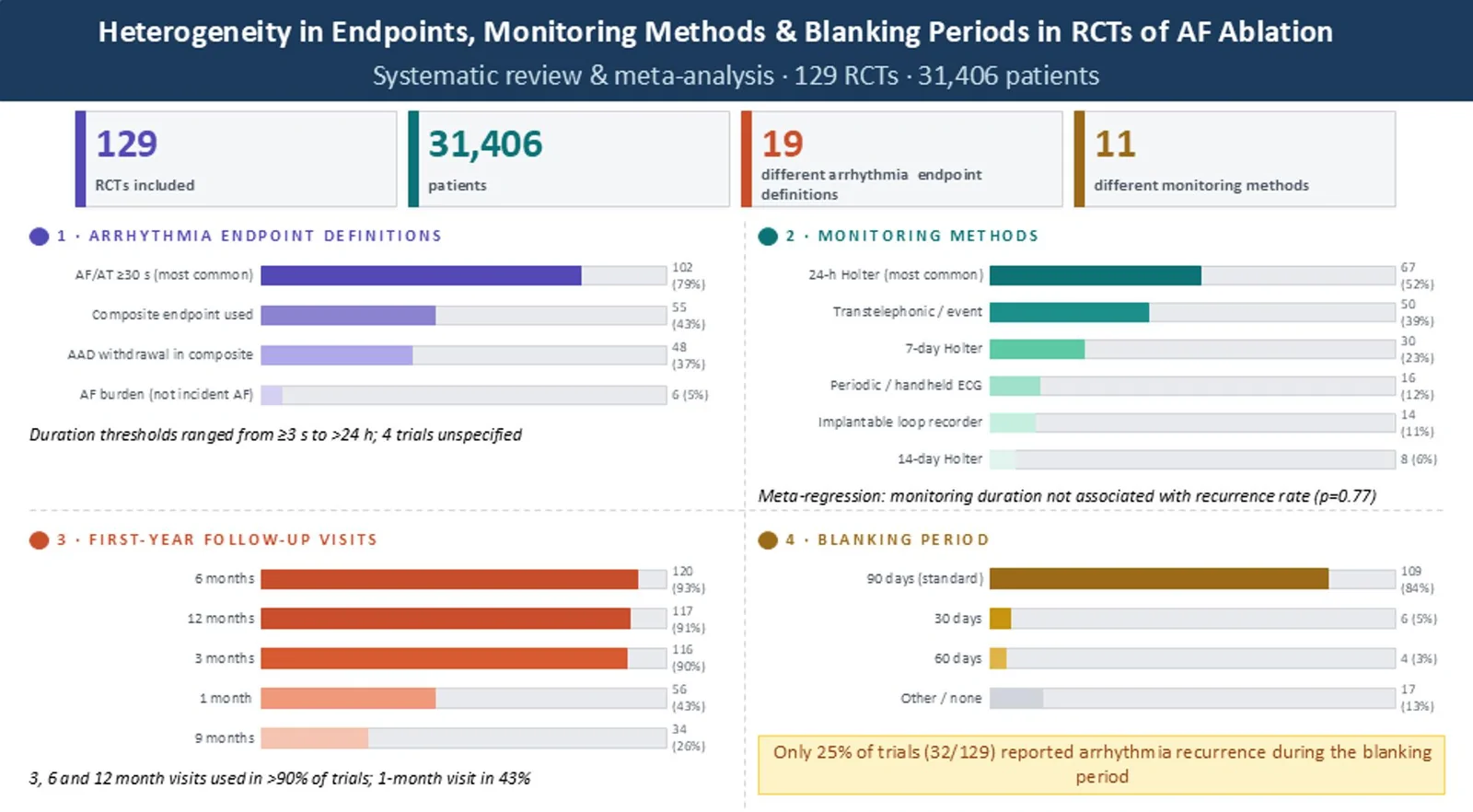

**Supplementary Information:**

The online version contains supplementary material available at https://doi.org/10.1007/s10840-026-02388-6.

## Introduction

Catheter ablation for atrial fibrillation (AF), predominantly through isolation of the pulmonary veins (PVs), is a cornerstone of rhythm control. Guidelines recommend catheter ablation in patients with symptomatic drug-refractory AF and in patients with tachycardia-mediated cardiomyopathy [1, 2]. This has conventionally been performed using thermal technology including radiofrequency (RF) or cryoballoon. However, the advent of pulsed field ablation (PFA) which relies on cell electroporation has shown similar efficacy compared to thermal ablation with improved procedural workflow and a different safety profile [3–5].

Randomised controlled trials (RCTs) remain the mainstay evidence that clinicians rely on to understand and compare clinical outcomes concerning AF ablation. While the number of RCTs being performed is growing, challenges arise when interpreting clinical outcomes which are heavily influenced by individual study design. Trials might differ with regards to arrhythmia endpoint definition and inclusion of other clinical outcomes such as antiarrhythmic drug (AAD) use, repeat ablation and cardioversion into a composite end point. Furthermore, detection of atrial tachyarrhythmias is well-recognised to increase with prolonged cardiac monitoring [6]. Yet there is heterogeneity in the method of arrhythmia monitoring and frequency which affects rate of reported recurrence. Furthermore, most trials include a blanking period post-ablation to account for an initial inflammatory effect from ablation and lesion maturation. However, accumulating data suggest that early arrhythmia recurrence is associated with late recurrence and higher AF burden after ablation [7, 8]. Most data regarding the pro-inflammatory effects were in the context of thermal ablation and it is unknown whether similar effects are observed with PFA.

In this systematic review and meta-analysis, we sought to evaluate the heterogeneity in RCT study designs in catheter ablation for AF by evaluating endpoints of arrhythmia recurrence, method of arrhythmia monitoring, schedule of follow-up and the use of a blanking period.

## Methods

### Search strategy

We searched electronic databases Embase and Ovid MEDLINE from database inception to August 2025. Medical Subject Headings (MeSH) keywords and other common designations for ‘atrial fibrillation’ and different catheter ablation modalities, including ‘catheter ablation’, ‘radiofrequency ablation’, ‘cryoablation’ and ‘pulsed field ablation’ were combined. The full line by line search strategy can be found in Table S1 of the Data Supplement. The search was performed in accordance with the Preferred Reporting Items for Systematic Reviews and Meta-Analyses (PRISMA) guidelines and the study was registered with the international PROSPERO registry (CRD420251118260). The list of included studies and references for them can be found in the Data Supplement.

### Eligibility and study selection

Eligible studies were parallel two-arm comparison RCTs on AF ablation published within the last 20 years, ≥ 100 participants, endpoints measuring the recurrence or burden of AF or atrial tachyarrhythmias, and ≥ 6 months of follow-up. Trials were excluded if they: (i) were not performed in humans, (ii) were not in English, (iii) involved surgical ablation procedures (including Maze or thoracoscopic ablation), (iv) follow-up or secondary analyses derived from primary trials, (v) compared the same ablation modality with different catheter tips, or power settings, or (vi) primarily evaluated the effects of repeat ablations.

### Data items and extraction

Two investigators (D.P & F.P.) extracted data, and a third investigator (F.J.H.) verified accuracy. For each RCT, we parsed the full text and supplementary materials to extract the year of publication, number of participants, ablation modality, inclusion criteria and patient demographics. We collected data on the arrhythmia endpoint definition used including AF or also other atrial tachyarrhythmias (atrial flutter [AFL] and atrial tachycardia [AT]), duration of atrial tachyarrhythmia that was considered a recurrence, and other clinical endpoints including subsequent direct current cardioversion (DCCV), withdrawal of AADs, arrhythmia-related hospitalisation or repeat ablation that was considered part of a composite primary endpoint in the study. We also collected schedule of follow-up for assessment of arrhythmia recurrence and the modality of arrhythmia monitoring performed. Blanking period data were also recorded including duration of blanking period post-ablation and whether any arrhythmia recurrences were reported during the blanking period.

### Endpoints

We summarised the data across the following endpoints: (1) definition of the arrhythmia recurrence in the study primary end point, (2) inclusion of other clinical outcomes and whether it was a composite primary end point, (3) modality used to monitor for arrhythmia recurrence, (4) schedule of follow-up for assessment of arrhythmia recurrence and (5) blanking period applied in each study and whether arrhythmia recurrence during this timeframe was reported.

### Statistical analyses

Meta-regression analyses were performed to evaluate the role of persistent AF in affecting arrhythmia recurrence across RCTs. Percentage number of patients with persistent AF were calculated across trials and meta-regression undertaken in a random-effects model. Additionally, a meta-regression was also undertaken to understand the role of type/duration of arrhythmia monitoring method and association with arrhythmia recurrence. All analyses were performed using Stata MP 19.0 (StataCorp LP, College Station, TX, United States).

## Results

### Study selection

**Fig. 1:**
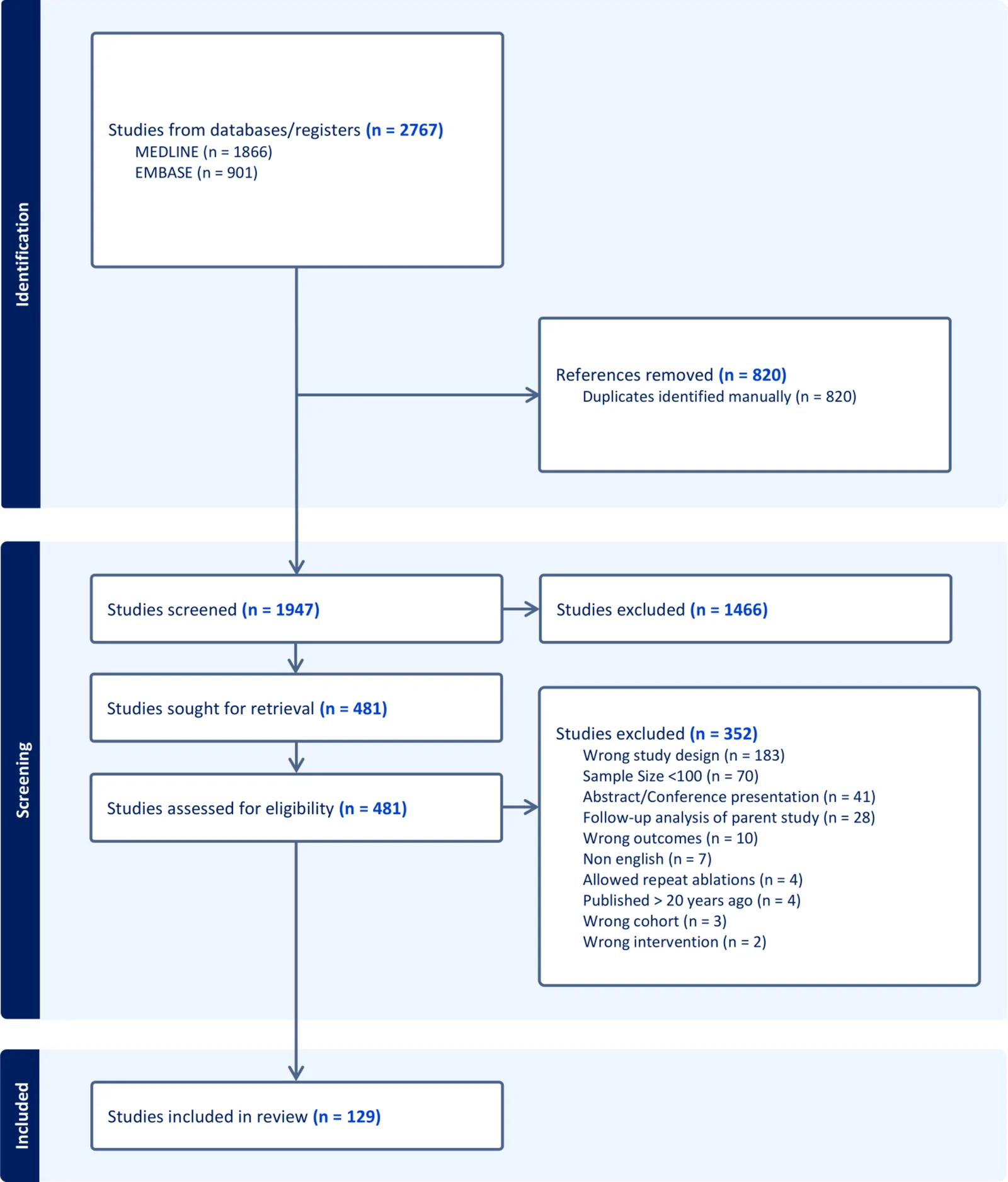
PRISMA flow diagram of study selection

Digital search yielded 2,767 studies of which 129 RCTs met inclusion criteria and were included in the final review after screening (Fig. 1). Across these trials, a total of 31,406 participants were enrolled.

### Characteristics of included trials

Modalities of ablation included RF (114 trials), cryoballoon (32 trials), laser balloon ablation (3 trials) and PFA (2 trials). In total, 57% (*n* = 17,841) of enrolled patients had paroxysmal AF and 43% (*n* = 13,565) had persistent AF. Total duration of follow-up ranged from 6 to 60 months. Trial characteristics are shown in Table 1 and Table S2 in the Data Supplement.

**Table 1:** Characteristics of included randomised controlled trials

Trial characteristics	Number of trials (*n* = 129)	Number of patients (*n* = 31,406)
Type of atrial fibrillation		
Paroxysmal	91	17,841
Persistent	77	13,565
Type of ablation		
Radiofrequency	114	23,221
Cryoballoon	32	4,368
Laser Balloon	3	335
Pulsed field	2	405
Longest Follow-up time (months)		
6	5	876
12	80	19,417
24	9	2,014
36	4	763
48	1	110
60	5	3,775

### Arrhythmia recurrence end point

**Fig. 2:**
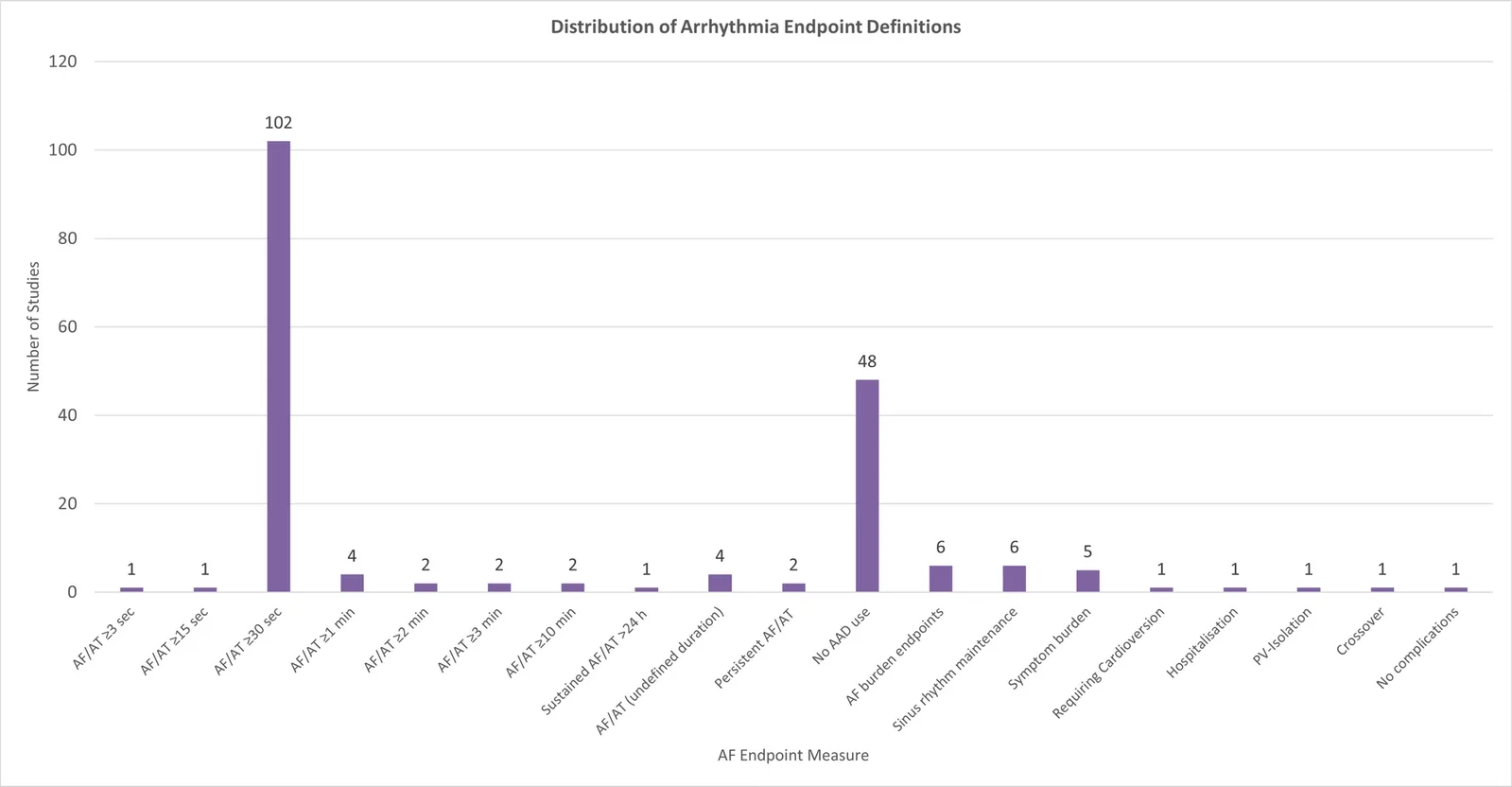
Distribution of arrhythmia endpoint definitions among randomised controlled trials. AAD, Antiarrhythmic drug; AF, atrial fibrillation; AT, atrial tachycardia; PV, Pulmonary vein

There were 19 different definitions of the primary arrhythmia recurrence endpoint across trials (Fig. 2 and Table S3 in the Data Supplement). A composite endpoint was used in 55 studies (43%) while an arrhythmia-only endpoint was used in 74 studies (57%). The most frequent criteria for the duration of atrial tachyarrhythmia was ≥ 30 s in 102 studies (79%). Other thresholds of AF/AT durations included ≥ 3 s (1 study), ≥ 15 s (1 study), ≥ 1 min (4 studies), ≥ 2 min (2 studies), ≥ 3 min (2 studies), and ≥ 10 min (2 studies). A sustained AF/AT duration of > 24 h was used in 1 study. No duration threshold for AF/AT was specified in 4 studies. In 6 trials, AF burden was reported instead. Additional clinical endpoints in the composite endpoint included withdrawal of AADs (48 trials), sinus rhythm maintenance (6 trials), symptom burden (5 trials), cardioversion requirement (1 trial), hospitalisation (1 trial), PV isolation failure (1 trial), crossover (1 trial) and no complications (1 trial). Meta-regression analysis showed that increased percentage of patients with persistent AF was associated with increased arrhythmia recurrence rate across trials (coefficient 0.34, 95% confidence interval 0.11–0.57, *p* = 0.003; Figure S1 in Data Supplement).

### Modality of arrhythmia monitoring

**Fig. 3:**
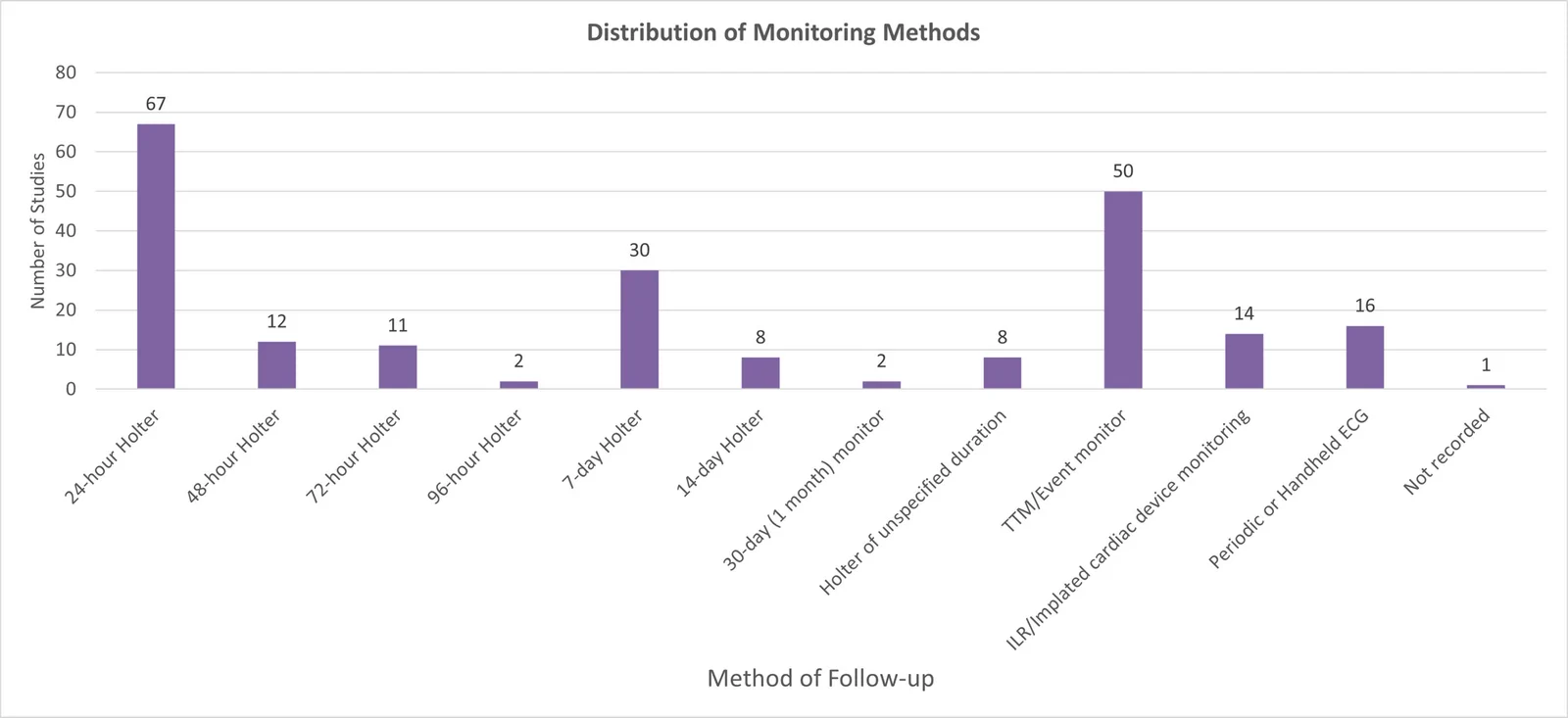
Distribution of method of monitoring among randomised controlled trials

There were 11 different methods and duration of monitoring for arrhythmia recurrence used across trials (Fig. 3 and Table S4 in the Data Supplement). Several studies included multiple methods of monitoring. 24‑hour Holter (67 trials; 52%) was the most common modality used to monitor arrhythmia recurrence. This was followed by trans‑telephonic/event‑triggered monitoring (50 trials; 39%), implantable loop recorder (ILR) (14 trials; 11%) and 7‑day Holter (30 trials; 23%). Other less common monitoring methods of arrhythmia monitoring included 48‑hour Holter (12 trials; 9%), 72‑hour Holter (11 trials; 9%), 96‑hour Holter (2 trials; 2%), 14‑day Holter (8 trials; 6%), 30‑day monitor (2 trials; 2%), periodic or handheld ECG (16 trials; 12%). Eight trials did not specify Holter duration, and one trial did not record the monitoring modality.

### Arrhythmia recurrence assessment during first year follow-up

**Fig. 4:**
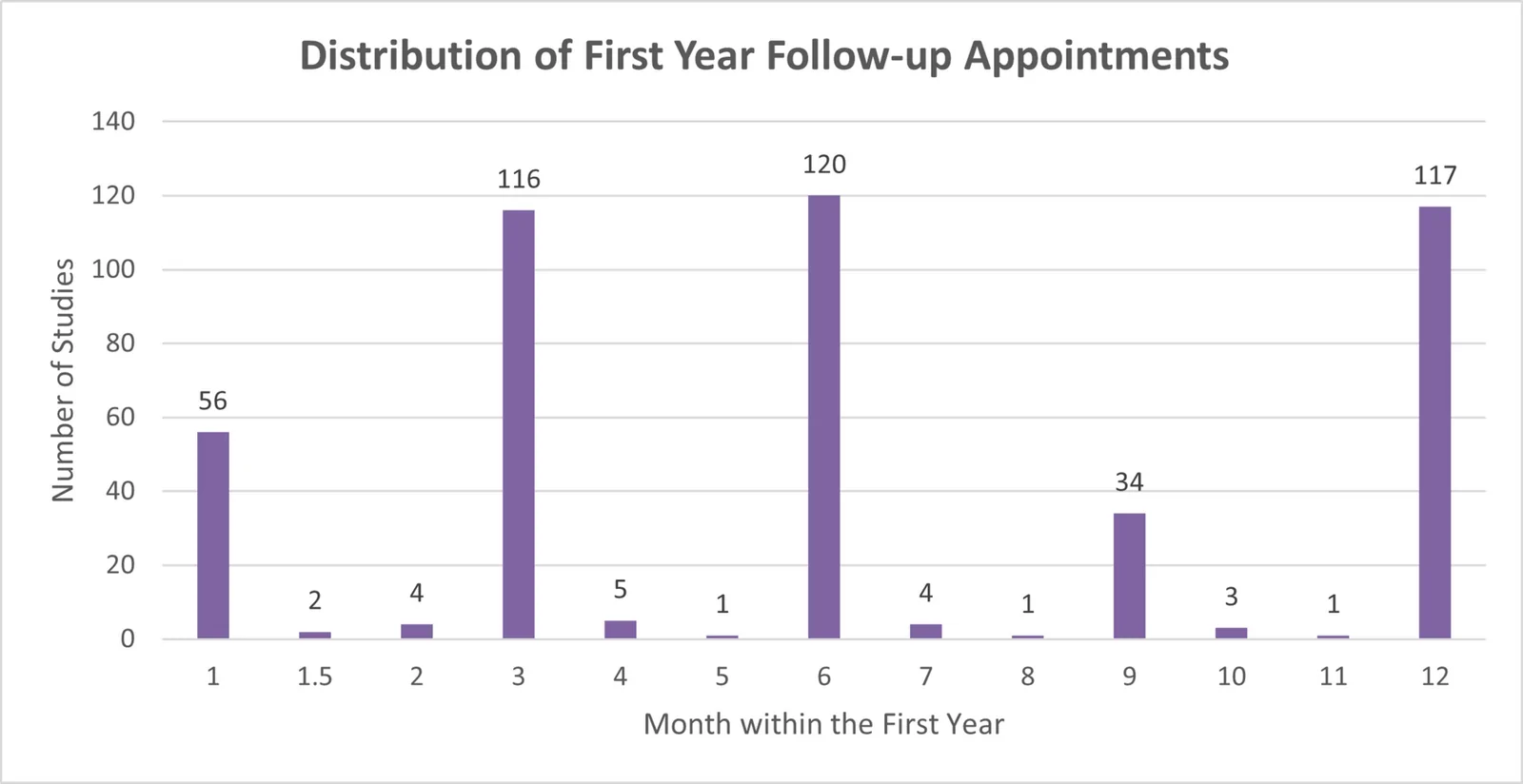
Distribution of follow-up appointments within the first-year post AF ablation

Assessment of arrhythmia recurrence in the first-year post-ablation in patients without an implantable cardiac monitor (ICM) was mostly performed at 3 months (116 trials; 90%), 6 months (120 trials; 93%), and 12 months (117 trials; 91%) (Fig. 4). A follow-up review at 1 month was conducted in 56 trials (43%) and an interval review at 9 months was conducted in 34 trials (26%). Other follow-up reviews post-ablation were conducted at 1.5 months (2 trials), 2 months (4 trials), 4 months (5 trials), 5 months (1 trial), 7 months (4 trials), 10 months (3 trials), 11 months (1 trial).

### Blanking periods

**Fig. 5:**
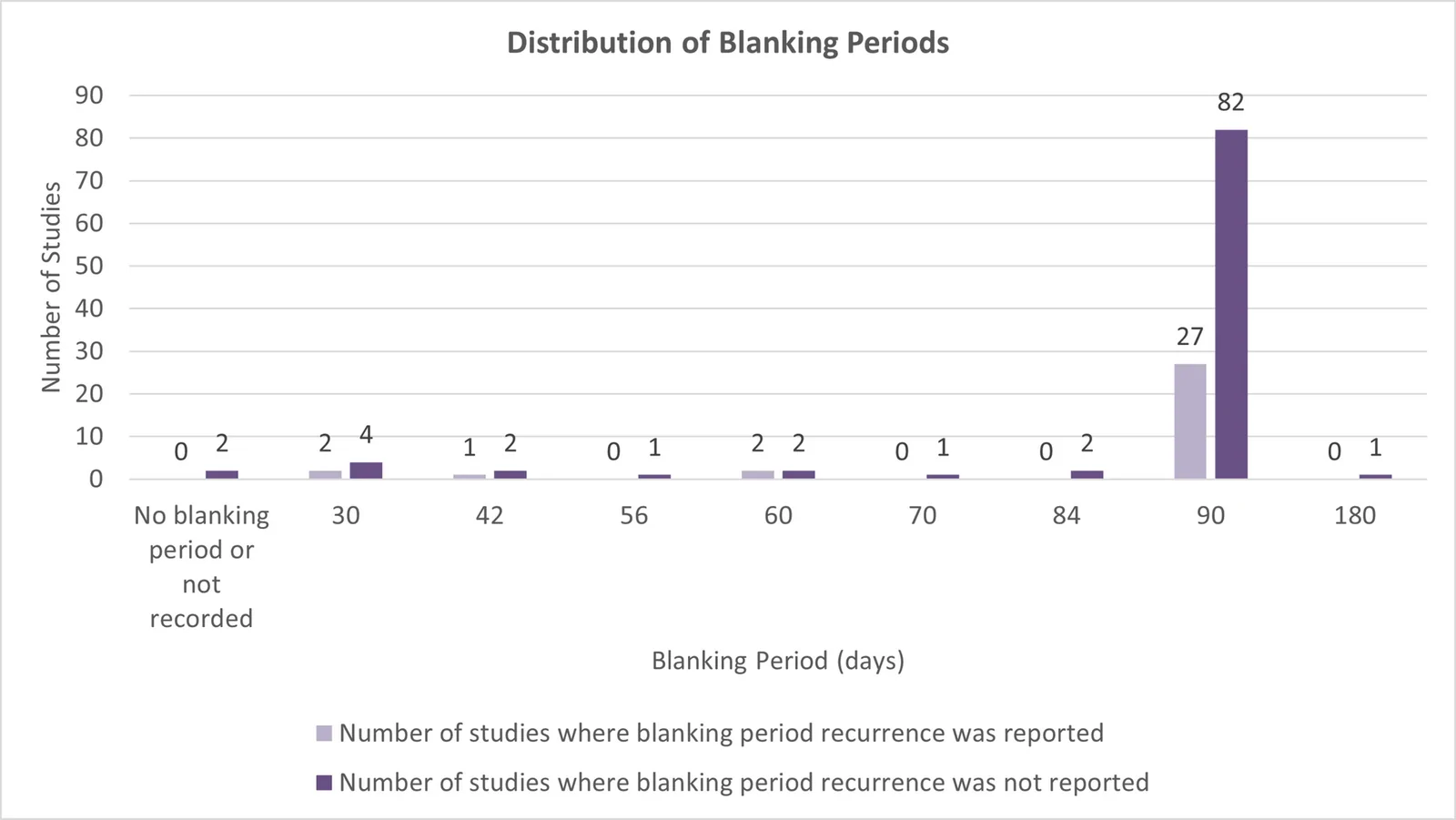
Distribution of blanking period length and reporting of arrhythmia recurrence during the blanking period

**Fig. 6:**
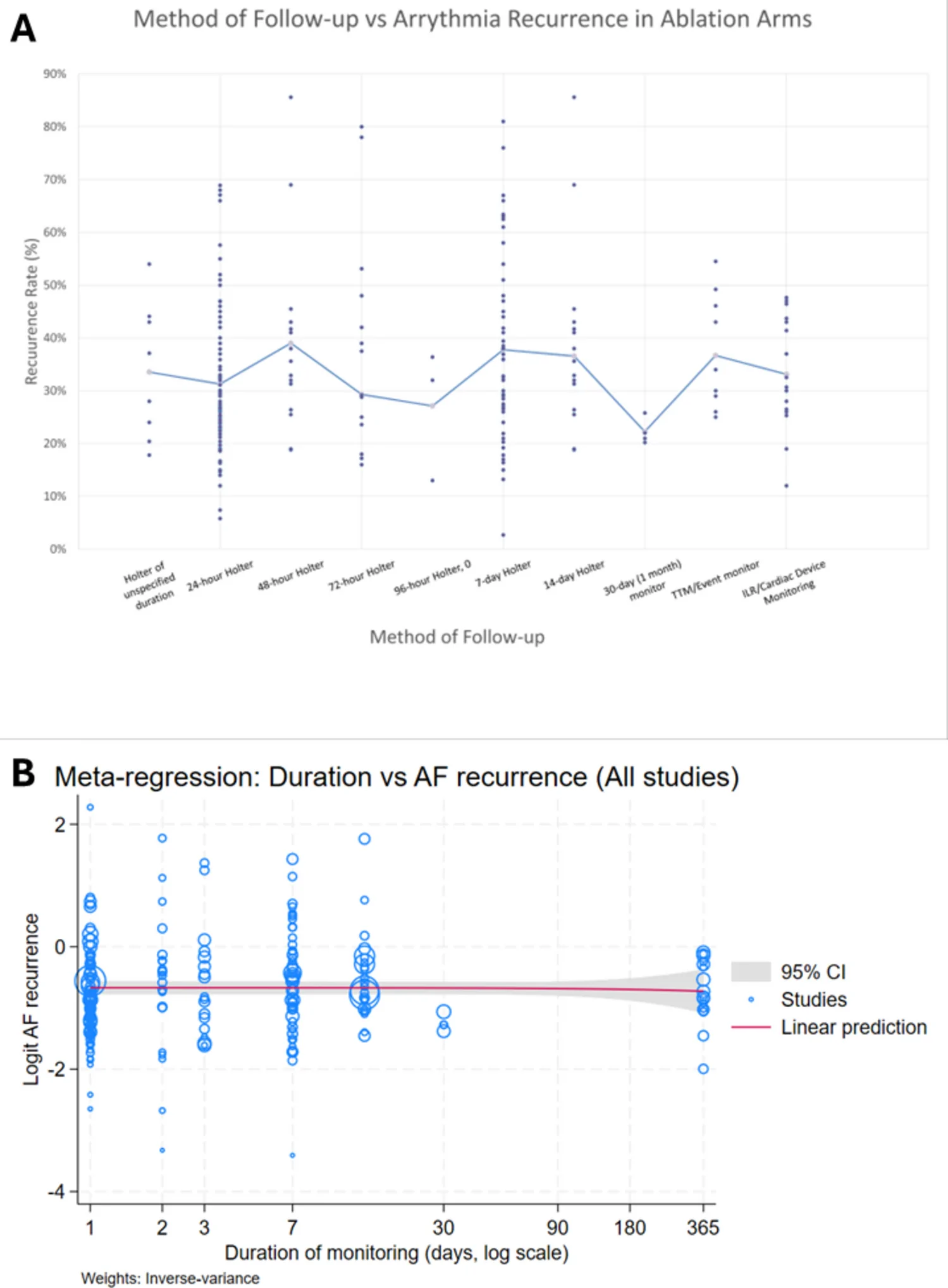
(**A**) Arrhythmia recurrence rate according to monitoring method. (**B**) Meta-regression analysis of duration of method of monitoring and rate of arrhythmia recurrence. Solid line denotes the monitoring method mean recurrence rate

The blanking period where arrhythmia recurrence was not considered failure post-ablation was predominantly 90 days (109 trials; 84%) (Fig. 5). One trial did not apply a blanking period and one trial did not report one, 6 trials had a 30-day blanking period, 4 trials had a 60-day blanking period and 1 trial had a 180-day blanking period. Reporting of any arrhythmia recurrence during the blanking period was reported in 32 trials (25%). On meta-regression analysis, duration of method of arrhythmia monitoring was not significantly associated with rate of atrial tachyarrhythmia recurrence (*p* = 0.77; Fig. 6). This remained non-significant when meta-regression analyses were performed separately for RCTs of patients with paroxysmal AF only (*p* = 0.30) or persistent AF (*p* = 0.43).

## Discussion

We performed a systematic review and meta-analysis of RCTs of catheter ablation for AF to evaluate arrhythmia endpoint definitions, method of monitoring, schedule of follow-up and the application of a blanking period. We found that amongst 129 RCTs with 31,406 patients.

1. There were 19 different arrhythmia end point definitions used across trials with the most common being AF/atrial tachyarrhythmia lasting 30 s or longer (79%)
2. There were eleven different methods of arrhythmia monitoring with the most common being 24-h Holter (52%) followed by trans-telephonic/event-triggered monitoring (39%)
3. Arrhythmia recurrence was most commonly assessed at a schedule of 3 months, 6 months and 12 months (> 90% of trials)
4. Most trials applied a blanking period of 90 days post-ablation (84%) and any arrhythmia recurrences during the blanking period were reported in only one-quarter (25%) of trials

### Arrhythmia endpoint

Randomised controlled trials provide a crucial level of evidence which guide and shape clinical practice. Nevertheless, there are inherent limitations to all trial designs including the designated trial primary endpoint. We observed heterogeneity in the primary arrhythmia endpoint and the definition of arrhythmia recurrence across AF ablation trials. Although most commonly this was atrial tachyarrhythmia lasting ≥ 30 s, there were 19 different arrhythmia endpoint definitions. This included different threshold durations for atrial tachyarrhythmia (between ≥ 3 s to ≥ 10 min), the use of AF burden (6 trials) as opposed to incident atrial tachyarrhythmia, and the inclusion of other clinical endpoints as part of a composite primary endpoint in 55 trials. Thus the pre-ablation counselling provided to our patients must account for arrhythmia endpoint definition for what constitutes a ‘success’ post-ablation. Additionally, there is limited relevance of a brief atrial tachyarrhythmia recurrence detected on routine monitoring in an otherwise asymptomatic patient post-ablation particularly if the overall burden of AF is low. An analysis of the CIRCA-DOSE study showed that patients with AF recurrences lasting less than 1 h and an overall AF burden ≤ 0.1% as detected by ICM had comparable healthcare utilisation to patients without recurrences [9]. AF burden remains clinically relevant whereby quality-of-life is inversely correlated with daily AF burden post-ablation [10]. Implantable cardiac monitors remain the gold standard in detection of post-ablation atrial arrhythmias in trial settings as short-duration monitors (24 h/48 h) having poor sensitivity (15.8–24.5%) for detection of arrhythmias although 14-day electrocardiogram monitoring tends to have a more comparable sensitivity (64.6%) [11]. While a more uniform definition of arrhythmia recurrence would certainly benefit trial interpretation, improve comparability between trials and aids clinical practice, the challenge remains what the ideal endpoint would be and is contingent on monitoring method and follow-up frequency.

### Method of arrhythmia monitoring

The method of arrhythmia monitoring including modality and duration was highly variable in trials. Most trials used either TTM/event-triggered monitoring or a 24-h Holter monitor while only fourteen (11%) trials used an ICM. It is well-recognised that a longer duration of monitoring is more likely to detect cardiac arrhythmias [6]. However, an ICM is not standard-of-care post-ablation in routine clinical practice and the financial constraints even in the context of a clinical trial may be prohibitive. While it appears feasible to perform a standard duration Holter monitor (usually 24 h) in a trial follow-up protocol, this remains a relatively short duration of monitoring compared with an ICM and is limited in sensitivity. Instead, the increasing ubiquity of wearable technology and its improving accuracy for the detection of AF could be a feasible modality moving forward [12, 13]. A pragmatic clinical trial in combination with regular follow-up schedule (e.g. 3, 6 and 12 months as most commonly adopted across trials) would better reflect real-world clinical practice and improve comparability of results between trials.

### Blanking period post-ablation

The role of the blanking period post-ablation and its duration is being increasingly questioned. The blanking period was initiated because of acute inflammatory changes that may predispose to a transient increased in post-procedural atrial tachyarrhythmias. The most commonly held period of 90 days is derived empirically. Yet, accumulating data suggest that early atrial arrhythmia recurrence is strongly associated with later recurrences [7, 14]. A study that prospectively enrolled patients with AF using thermal ablation reported an optimal blanking period of three weeks for the most accurate correlation between early and late arrhythmia recurrence (area under the curve 0.7) [15]. Current guidelines suggest a blanking period of 8 weeks to minimise misclassification of early arrhythmia recurrence and reduce potentially unnecessary repeat ablation procedures [16]. The advent of PFA, with a different mechanism of achieving electrical isolation through cell electroporation, may not have the same inflammatory profile compared with conventional thermal ablation. Indeed, a retrospective study has shown that only recurrence in the second or third month after PFA predicted late recurrences as compared with recurrences in the first month [17]. While an initial blanking period may be reasonable in avoiding an early repeat ablation procedure, it should be acknowledged that for patients, symptomatic recurrences, even early and especially when requiring hospitalisation or DCCV, is still significant. Furthermore, any blanking period inherently favours catheter ablation over medical therapy with regards to arrhythmic or clinical efficacy. At present, only one-quarter of RCTs report arrhythmia recurrence during the blanking period. It would be pertinent that arrhythmic recurrence even during the blanking period should be routinely reported in clinical trials as well.

### Endpoint recommendation

Ideally, a combination of daily metrics such as through consumer smartphone-based photoplethysmography signals and 14-day monitoring for AF burden would feasibly capture relevant atrial arrhythmia recurrence, recognising an AF duration of > 1 h and an AF burden of > 0.1% as clinically relevant [18, 19]. Beyond a lone arrhythmia endpoint, the inclusion of other clinically significant outcomes would be insightful and relevant for patients; several trials included the withdrawal of AADs, repeat ablation and DCCVs. The use of co-primary endpoints could be considered in trial design, that being an arrhythmia endpoint and a clinical endpoint. Quality-of-life metrics are important patient-centred endpoints that remain essential to evaluating procedural success [9, 20, 21].

### Novel endpoints

Beyond arrhythmia recurrence, be that incident arrhythmias or AF burden, as well as quality-of-life and healthcare utilisation endpoints, there are other potential secondary endpoints that could be considered in trials. A potentially novel endpoint is progression of AF, whereby radiofrequency ablation has been associated with reduced progression of paroxysmal to persistent AF compared with cryoablation [22]. When interpreting clinical efficacy of catheter ablation, an understanding of lesion durability and transmurality is important especially with our growing understanding of PFA technology. Pulmonary vein reconnection and posterior wall reconnection has been noted in up to 57% and 55% of patients with symptomatic arrhythmia recurrences after PFA, respectively [23]. Future trials could also consider other structurally mechanistic endpoints such as change in left atrial volume, fibrosis burden on cardiac magnetic resonance imaging with late gadolinium enhancement and other markers of structural remodelling to better understand drivers of disease progression [24, 25].

### Limitations

We acknowledge several limitations in this study. First, this systematic review concerns RCTs that performed catheter ablation for AF. These inherently reflect higher quality studies although by nature, may not be easily extrapolated to real-world context depending on the individual trial design. The arrhythmia endpoints, monitoring methods and blanking periods adopted in single-arm studies or non-randomised cohort studies are not reflected here. Second, adjunctive ablation strategies remain heterogeneous between trials such as posterior wall ablation, superior vena cava isolation and linear lesions which could limit interpretability of clinical efficacy within each trial. Nevertheless, this was not the aim of the present systematic review which focuses primarily on the trial design pertaining to endpoints, rhythm monitoring method and follow-up. Third, we included clinical trials over the last 20-year period. Patient selection, type of anticoagulation (vitamin K antagonists to direct oral anticoagulants) and procedural characteristics have significantly changed during this period and should be acknowledged in the design of the included trials. In regard to patient selection specifically, earlier trials generally enrolled patients with paroxysmal AF with minimal comorbidities. However, more contemporary trials in the era of PFA are increasingly enrolling older patients with more persistent AF and more comorbidities [26, 27]. These are a different set of patients compared with earlier trials and may have implications for trial design such as risk of procedural-related complications in older patients [28], degree of healthcare utilisation such as heart failure hospitalisation, and patients’ ability to undertake certain forms of rhythm monitoring such as smartphone plethysmography or onerous prolonged ambulatory monitoring. Fourth, we did not include surgical AF ablation in this study as this approach is different to endocardial catheter ablation and the defined endpoints include safety endpoints and blanking periods would differ.

## Conclusions

In this systematic review of RCTs of catheter ablation for AF, there was significant heterogeneity in the arrhythmia endpoints (19 different definitions) and method of arrhythmia monitoring (11 different methods) across trials. Most RCTs used a 90-day blanking period although arrhythmia recurrence in the blanking period was reported in only 25% of studies. There is need to develop a more uniform arrhythmia endpoint definition, modality of monitoring and the utility and duration of a blanking period post-ablation to improve comparability between trials and real- world application.

## Supplementary Information

Below is the link to the electronic supplementary material.

Supplementary file1 (DOCX 202 KB)

## Data Availability

Data available upon request.

## Abbreviations

AF: Atrial fibrillation
RCT: Randomized controlled trial
AAD: Antiarrhythmic drug
AT: Atrial tachyarrhythmia
RFA: Radiofrequency ablation
CBA: Cryoballoon ablation
LBA: Laser balloon ablation
PFA: Pulsed field ablation
ICM: Implantable cardiac monitor
