# Supplementary material for: Heterogeneity in endpoints, monitoring methods and blanking periods in clinical trials of atrial fibrillation ablation: a systematic review and meta-analysis
Source: J Interv Card Electrophysiol. 2026 Jun 27;69(9):2779–88. doi: 10.1007/s10840-026-02388-6 (PMC13769684; doi:10.1007/s10840-026-02388-6)
Supplement: Supplementary file 1 [file 10840_2026_2388_MOESM1_ESM.docx]

**Heterogeneity in Endpoints, Monitoring Methods and Blanking Periods in Clinical Trials of Atrial Fibrillation Ablation: A Systematic Review and
Meta-Analysis**

**Data Supplement**

**Table S1. Literature Search Strategy**

Embase Classic + Embase from 1947 to September 26^th^, 2025

| Search Number | Query |
| --- | --- |
| 1 | exp atrial fibrillation/ |
| 2 | exp atrial fibrillations/ |
| 3 | 1 or 2 |
| 4 | exp catheter ablation/ |
| 5 | exp radiofrequency ablation/ |
| 6 | exp cryoablation/ |
| 7 | exp radio-frequency ablation/ |
| 8 | exp radio frequency ablation/ |
| 9 | pulsed field ablation.mp. [mp=title, abstract, heading word, drug trade name, original title, device manufacturer, drug manufacturer, device trade name, keyword heading word, floating subheading word, candidate term word] |
| 10 | PFA.mp. [mp=title, abstract, heading word, drug trade name, original title, device manufacturer, drug manufacturer, device trade name, keyword heading word, floating subheading word, candidate term word] |
| 11 | percutaneous catheter ablation.mp. [mp=title, abstract, heading word, drug trade name, original title, device manufacturer, drug manufacturer, device trade name, keyword heading word, floating subheading word, candidate term word] |
| 12 | 4 or 5 or 6 or 7 or 8 or 9 or 10 or 11 |
| 13 | 3 and 12 |
| 14 | limit 13 to randomized controlled trial |

Ovid MEDLINE from 1946 to September 26^th^, 2025

| Search Number | Query |
| --- | --- |
| 1 | exp atrial fibrillation/ |
| 2 | exp atrial fibrillations/ |
| 3 | 1 or 2 |
| 4 | exp catheter ablation/ |
| 5 | exp radiofrequency ablation/ |
| 6 | exp cryoablation/ |
| 7 | exp radio-frequency ablation/ |
| 8 | exp radio frequency ablation/ |
| 9 | pulsed field ablation.mp. [mp=title, book title, abstract, original title, name of substance word, subject heading word, floating sub-heading word, keyword heading word, organism supplementary concept word, protocol supplementary concept word, rare disease supplementary concept word, unique identifier, synonyms, population supplementary concept word, anatomy supplementary concept word] |
| 10 | PFA.mp. [mp=title, book title, abstract, original title, name of substance word, subject heading word, floating sub-heading word, keyword heading word, organism supplementary concept word, protocol supplementary concept word, rare disease supplementary concept word, unique identifier, synonyms, population supplementary concept word, anatomy supplementary concept word] |
| 11 | percutaneous catheter ablation.mp. [mp=title, book title, abstract, original title, name of substance word, subject heading word, floating sub-heading word, keyword heading word, organism supplementary concept word, protocol supplementary concept word, rare disease supplementary concept word, unique identifier, synonyms, population supplementary concept word, anatomy supplementary concept word] |
| 12 | 4 or 5 or 6 or 7 or 8 or 9 or 10 or 11 |
| 13 | 3 and 12 |
| 14 | limit 13 to randomized controlled trial |

**Table S2. Study Size, Ablation Modality and Patient Demographics**

| **First Author** | **Year Published** | **Total # of pts** | **Ablation modality** | **# of females** | **% Female** | **Average Age Treatment** | **Average Age Control** | **BMI Average Treatment** | **BMI Average Control** | **Diabetes Treatment (%)** | **Diabetes Control (%)** | **HTN Treatment (%)** | **HTN Control (%)** | **Prior Stroke/TIA Treatment (%)** | **Prior Stroke/TIA Control (%)** | **LA Diameter Treatment (mm)** | **LA Diameter Control (mm)** | **LVEF Treatment** | **LVEF Control** |
| --- | --- | --- | --- | --- | --- | --- | --- | --- | --- | --- | --- | --- | --- | --- | --- | --- | --- | --- | --- |
| Ahn | 2023 | 100 | CBA | 23.0 | 23.0 | 58.9 | 61.8 | 24.8 | 24.6 | 24.0 | 20.0 | 62.0 | 54.0 | NR | NR | 38.8 | 39.9 | 61.4 | 62.5 |
| Ahn | 2022 | 100 | CBA | 16.0 | 16.0 | 65.1 | 65.9 | 26.4 | 25.8 | 46.0 | 40.0 | 76.0 | 90.0 | NR | NR | 48.1 | 48.5 | 57.6 | 58.5 |
| Al-Kaisey | 2023 | 100 | RFA | 31.0 | 32.0 | 58.0 | 60.0 | 30.0 | 31.0 | 6.0 | 4.0 | 53.0 | 55.0 | 8.0 | 2.0 | NR | NR | NR | NR |
| Andrade | 2021 | 303 | CBA | 89.0 | 29.0 | 57.7 | 59.5 | 30.9 | 29.7 | NR | NR | 37.0 | 37.0 | 3.0 | 3.0 | 39.5 | 38.1 | 59.6 | 59.8 |
| Arbelo | 2014 | 120 | RFA | 36.0 | 29.3 | 55.0 | 55.0 | NR | NR | 3.4 | 6.6 | 32.2 | 39.3 | 1.7 | 0.0 | 41.0 | 41.0 | 62.0 | 62.0 |
| Arentz | 2007 | 110 | RFA | 27.0 | 25.0 | 55.0 | 56.0 | NR | NR | NR | NR | NR | NR | NR | NR | 40.0 | 40.0 | NR | NR |
| Ariyaratnam | 2025 | 100 | RFA | 31.0 | 31.0 | 57.9 | 60.5 | 29.8 | 30.1 | 8.3 | 13.5 | 68.8 | 57.7 | 12.5 | 3.8 | 39.9 | 41.1 | 59.9 | 61.3 |
| Aryana | 2021 | 110 | CBA, RFA | 45.0 | 41.0 | 70.0 | 67.0 | 29.0 | 30.0 | 27.0 | 25.0 | 67.0 | 64.0 | 7.0 | 7.0 | 44.0 | 44.0 | 61.0 | 60.0 |
| Baek | 2021 | 170 | RFA | 50.0 | 29.0 | 58.3 | 60.2 | NR | NR | 22.0 | 24.0 | 59.0 | 51.0 | 15.0 | 17.0 | 44.9 | 44.7 | 60.0 | 60.1 |
| Baimbetov | 2022 | 100 | CBA, RFA | 40.0 | 40.0 | 61.3 | 61.6 | NR | NR | NR | NR | NR | NR | NR | NR | 41.0 | 39.0 | 59.0 | 54.0 |
| Blomström-Lundqvist | 2019 | 155 | RFA, CBA | 35.0 | 22.6 | 55.8 | 56.3 | 27.3 | 26.9 | 3.8 | 3.9 | 46.8 | 30.3 | 5.1 | 0.0 | 41.7 | 41.7 | 56.1 | 56.2 |
| Brachmann | 2025 | 350 | RFA | 97.0 | 28.0 | 64.2 | 62.7 | 31.2 | 31.1 | NR | NR | NR | NR | NR | NR | 48.1 | 45.6 | 53.8 | 54.5 |
| Buist | 2018 | 269 | CBA, RFA | 78.0 | 29.0 | 59.7 | 58.2 | 27.8 | 27.1 | 10.6 | 6.6 | 43.2 | 38.5 | 6.0 | 7.4 | NR | NR | NR | NR |
| Bänsch | 2013 | 107 | RFA | 36.0 | 34.0 | 59.6 | 61.6 | NR | NR | 13.0 | 9.0 | 80.0 | 85.0 | 4.0 | 15.0 | 51.0 | 49.0 | 53.0 | 53.1 |
| Cao | 2025 | 248 | RFA | 90.0 | 36.0 | 67.0 | 62.5 | 25.5 | 26.1 | 9.0 | 15.0 | 47.0 | 51.0 | 17.0 | 16.0 | 40.0 | 40.0 | 55.0 | 56.0 |
| Caponi | 2010 | 299 | RFA | 61.0 | 20.0 | 59.5 | 57.2 | NR | NR | NR | NR | NR | NR | NR | NR | NR | NR | NR | NR |
| Castro-Urda | 2025 | 100 | CBA | 34.0 | 34.0 | 66.0 | 63.5 | 25.9 | 26.5 | 5.0 | 4.0 | 19.0 | 21.0 | NR | NR | 38.9 | 37.6 | 60.0 | 60.0 |
| Chen | 2023 | 438 | RFA | 219.0 | 50.0 | 70.2 | 70.7 | 24.3 | 24.6 | 14.6 | 19.6 | 59.4 | 64.8 | 7.8 | 9.6 | 38.8 | 38.8 | 62.4 | 62.4 |
| Choi | 2022 | 178 | RFA | 47.0 | 26.0 | 62.9 | 61.3 | NR | NR | 27.0 | 18.0 | 74.0 | 69.0 | 4.0 | 6.0 | 43.4 | 42.6 | 56.2 | 57.0 |
| Chun | 2021 | 200 | CBA, LBA | 88.0 | 44.0 | 65.0 | 66.5 | 28.3 | 28.0 | 12.0 | 9.0 | 65.0 | 68.0 | 2.0 | 4.0 | 39.1 | 39.8 | 61.5 | 61.5 |
| Corrado | 2010 | 320 | RFA | 77.0 | 24.0 | 55.0 | 57.0 | NR | NR | NR | NR | 87.0 | 63.0 | NR | NR | 45.0 | 46.0 | 54.0 | 53.0 |
| Darkner | 2014 | 212 | RFA | 36.0 | 17.0 | 62.0 | 61.0 | 26.6 | 26.8 | 9.0 | 8.0 | 37.0 | 42.0 | 6.0 | 7.0 | 44.0 | 44.0 | 51.0 | 50.0 |
| Deisenhofer | 2025 | 370 | RFA | 77.0 | 21.0 | 66.4 | 64.9 | NR | NR | 18.0 | 16.0 | 63.0 | 60.0 | 8.0 | 7.0 | 44.6 | 45.7 | 55.3 | 54.3 |
| DiBiase | 2016 | 203 | RFA | 52.0 | 25.6 | 62.0 | 60.0 | 30.0 | 29.0 | 22.0 | 24.0 | 45.0 | 48.0 | NR | NR | 47.0 | 48.0 | 29.0 | 30.0 |
| Ding | 2022 | 204 | CBA | 83.0 | 41.0 | 60.9 | 60.7 | 25.7 | 25.7 | 15.7 | 14.7 | 52.9 | 46.1 | 7.8 | 9.8 | 38.3 | 39.1 | 60.9 | 60.0 |
| Dixit | 2008 | 105 | RFA | 29.0 | 28.0 | 57.0 | 57.0 | NR | NR | NR | NR | NR | NR | NR | NR | NR | NR | NR | NR |
| Dong | 2015 | 146 | RFA | 36.0 | 25.0 | 55.0 | 56.0 | 27.2 | 26.7 | 18.0 | 18.0 | 51.0 | 51.0 | NR | NR | 42.0 | 43.0 | 61.5 | 60.5 |
| Dukkipati | 2015 | 334 | LBA, RFA | 115.0 | 33.6 | 59.7 | 60.1 | NR | NR | 15.3 | 9.9 | 59.4 | 58.1 | 6.5 | 7.6 | 40.0 | 40.0 | 60.6 | 60.2 |
| Dulai | 2024 | 126 | CBA | 37.0 | 29.5 | 66.1 | 67.5 | 29.1 | 28.9 | 9.4 | 8.1 | 46.9 | 48.4 | 3.1 | 0.0 | 42.5 | 41.0 | 55.0 | 55.0 |
| Duytschaever | 2018 | 153 | RFA | 41.0 | 26.8 | 63.0 | 62.0 | 27.3 | 27.3 | 8.0 | 4.0 | 30.0 | 43.0 | 7.0 | 8.0 | 41.0 | 41.0 | NR | NR |
| Ejima | 2019 | 136 | RFA | 34.0 | 25.0 | 63.6 | 63.8 | 25.0 | 23.9 | 12.0 | 13.0 | 58.0 | 47.0 | 7.0 | 3.0 | 37.2 | 38.4 | 60.2 | 60.7 |
| Estner | 2011 | 116 | RFA | 30.0 | 26.0 | 56.8 | 59.4 | NR | NR | NR | NR | 56.0 | 58.0 | NR | NR | 49.0 | 47.0 | NR | NR |
| Falasconi | 2025 | 156 | RFA | 48.0 | 31.0 | 65.4 | 65.9 | 28.1 | 29.6 | 10.3 | 10.3 | 55.0 | 59.0 | NR | NR | 43.3 | 44.7 | 54.7 | 53.7 |
| Fassini | 2005 | 187 | RFA | 37.0 | 20.0 | 54.0 | 57.0 | NR | NR | NR | NR | NR | NR | NR | NR | 43.7 | 41.5 | 55.3 | 56.8 |
| Faustino | 2015 | 150 | RFA | 58.0 | 39.0 | 63.4 | 62.3 | 26.0 | 25.9 | 22.7 | 24.0 | 74.7 | 64.0 | NR | NR | 43.8 | 44.0 | 59.3 | 58.5 |
| Ferrero-de-Loma-Osorio | 2017 | 140 | CBA | 45.0 | 32.0 | 58.0 | 54.0 | 24.8 | 24.9 | 11.0 | 7.0 | 47.0 | 33.0 | 1.0 | 3.0 | NR | NR | 62.9 | 65.4 |
| Fiala | 2008 | 110 | RFA | 21.0 | 19.0 | 53.0 | 51.0 | NR | NR | NR | NR | 54.0 | 52.0 | NR | NR | 40.0 | 38.0 | 60.0 | 59.0 |
| Fichtner | 2013 | 207 | RFA | 61.0 | 29.5 | 59.4 | 60.9 | NR | NR | 6.6 | 7.0 | 65.7 | 56.0 | 15.2 | 13.0 | 43.5 | 44.3 | NR | NR |
| Fink | 2017 | 118 | RFA | 34.0 | 29.0 | 60.9 | 62.1 | 27.8 | 28.1 | 7.0 | 8.0 | 51.0 | 57.0 | NR | NR | 46.7 | 47.3 | NR | NR |
| Gaita | 2008 | 204 | RFA | 42.0 | 21.0 | NR | NR | NR | NR | NR | NR | NR | NR | NR | NR | NR | NR | NR | NR |
| Gal | 2014 | 460 | RFA | 113.0 | 24.6 | 56.1 | 56.6 | 27.4 | 27.9 | 7.4 | 5.7 | 30.9 | 39.3 | 3.9 | 7.0 | 40.6 | 41.7 | NR | NR |
| Gallagher | 2021 | 100 | CBA, RFA | 22.0 | 22.0 | 63.0 | 63.0 | 31.0 | 30.0 | 14.0 | 12.0 | 38.0 | 38.0 | 12.0 | 16.0 | 45.0 | 45.0 | 51.0 | 54.0 |
| Gula | 2016 | 119 | RFA | 27.0 | 22.0 | 59.7 | 57.1 | NR | NR | 8.5 | 3.3 | 45.8 | 38.3 | 13.6 | 5.0 | NR | NR | NR | NR |
| Inoue | 2021 | 512 | RFA | 121.0 | 24.0 | 66.0 | 67.0 | 24.0 | 24.0 | 19.0 | 15.0 | 59.0 | 58.0 | 10.0 | 8.0 | 42.0 | 42.0 | 58.0 | 59.0 |
| Jaïs | 2008 | 112 | RFA | 18.0 | 16.1 | 49.7 | 52.4 | NR | NR | 1.9 | 3.4 | 21.6 | 30.5 | 1.9 | 11.9 | 39.5 | 40.0 | 63.1 | 65.6 |
| Jiang | 2022 | 225 | RFA | 93.0 | 41.0 | 60.0 | 59.0 | 24.3 | 24.5 | 5.4 | 6.6 | 50.3 | 43.3 | 9.4 | 11.8 | NR | NR | 65.8 | 65.2 |
| Kaiser | 2024 | 100 | RFA | 31.0 | 31.0 | 65.2 | 67.5 | 29.8 | 27.6 | NR | NR | 59.0 | 73.0 | 10.0 | 14.0 | NR | NR | 53.4 | 50.3 |
| Kalman | 2023 | 89 | CBA, RFA | 26.0 | 29.0 | 58.0 | 59.0 | 30.0 | 30.0 | 6.0 | 2.0 | 54.0 | 54.0 | 6.0 | 0.0 | 47.0 | 44.0 | 57.0 | 61.0 |
| Kang | 2014 | 200 | RFA | 51.0 | 25.0 | 57.9 | 55.7 | NR | NR | 17.0 | 11.0 | 53.0 | 41.0 | 9.0 | 6.0 | 40.1 | 39.5 | 64.6 | 63.4 |
| Karch | 2005 | 100 | RFA | 36.0 | 36.0 | 59.0 | 61.0 | NR | NR | NR | NR | NR | NR | NR | NR | 47.0 | 46.0 | 64.0 | 62.0 |
| Kim | 2025 | 277 | CBA, RFA | 69.0 | 24.0 | 63.0 | 63.0 | 24.6 | 24.5 | 21.0 | 15.0 | 54.0 | 49.0 | 14.0 | 13.0 | 41.0 | 41.0 | 63.0 | 62.0 |
| Kim | 2010 | 102 | RFA | 20.0 | 20.0 | 52.3 | 54.2 | NR | NR | 6.0 | 13.0 | 33.0 | 32.0 | NR | NR | 39.0 | 40.0 | 54.0 | 56.0 |
| Kim | 2024 | 277 | CBA | 90.0 | 32.5 | 60.9 | 61.3 | 24.3 | 24.3 | 12.6 | 15.5 | 37.8 | 50.7 | 5.9 | 8.5 | 39.1 | 38.9 | 64.2 | 65.5 |
| Kircher | 2018 | 124 | RFA | 47.0 | 38.0 | 62.0 | 63.0 | NR | NR | NR | NR | 79.0 | 81.0 | NR | NR | 43.0 | 42.0 | 59.0 | 61.0 |
| Kisler | 2023 | 338 | RFA | 79.0 | 23.4 | 65.7 | 65.5 | 29.1 | 28.6 | 10.0 | 10.7 | 50.0 | 44.0 | 6.5 | 4.2 | 46.0 | 44.0 | 56.0 | 55.0 |
| Kobori | 2015 | 2113 | RFA | 534.0 | 25.3 | 58.6 | 68.5 | 24.3 | 23.7 | 12.7 | 14.1 | 47.6 | 58.9 | 5.9 | 10.8 | 38.9 | 39.2 | 64.3 | 64.6 |
| Kuck | 2016 | 762 | RFA, CBA | NR | NR | 60.1 | 59.9 | 27.8 | 28.0 | 5.9 | 9.9 | 58.8 | 57.5 | NR | NR | 40.6 | 40.8 | NR | NR |
| Kuck | 2021 | 255 | RFA | 148.0 | 58.0 | 67.8 | 67.6 | NR | NR | 10.2 | 11.0 | 93.8 | 96.9 | 9.4 | 6.3 | 42.1 | 43.4 | 61.8 | 62.3 |
| Kuck | 2019 | 140 | RFA | 14.0 | 10.0 | 65.0 | 65.0 | 29.4 | 28.4 | 35.0 | 31.0 | 82.0 | 76.0 | NR | NR | 50.0 | 51.0 | 27.8 | 24.8 |
| Kuniss | 2021 | 218 | CBA | 70.0 | 32.1 | 50.5 | 54.1 | NR | NR | 90.0 | 3.6 | 30.8 | 36.0 | 0.0 | 90.0 | 46.8 | 47.7 | 62.8 | 63.7 |
| Lee | 2018 | 500 | RFA | 127.0 | 25.0 | 55.5 | 56.0 | 24.7 | 24.8 | 10.0 | 6.0 | 44.0 | 39.0 | 3.2 | 5.6 | 38.8 | 39.1 | 56.8 | 56.3 |
| Lee | 2019 | 150 | RFA | 22.0 | 14.7 | 55.9 | 55.8 | 25.2 | 25.2 | 6.7 | 13.3 | 42.7 | 41.3 | 12.0 | 9.3 | 43.4 | 42.7 | 54.0 | 53.5 |
| Lee | 2019 | 207 | RFA | 35.0 | 17.0 | 58.9 | 58.6 | NR | NR | 12.7 | 17.1 | 43.1 | 50.5 | 9.8 | 12.4 | 45.0 | 44.5 | 59.2 | 58.8 |
| Lin | 2023 | 170 | RFA,CBA | 34.0 | 20.0 | 61.9 | 62.0 | 26.5 | 26.6 | 24.7 | 15.3 | 61.2 | 62.4 | 30.6 | 30.6 | 44.2 | 43.9 | 55.1 | 56.9 |
| Lin | 2019 | 142 | RFA | 54.0 | 38.0 | 63.2 | 63.1 | NR | NR | 12.7 | 7.0 | 56.3 | 46.5 | 15.5 | 9.9 | 43.4 | 44.4 | 61.4 | 61.2 |
| Lin | 2012 | 126 | RFA | 44.0 | 35.0 | 56.0 | 53.0 | NR | NR | 15.0 | 10.0 | 48.0 | 50.0 | NR | NR | 39.0 | 38.0 | 61.0 | 61.0 |
| Liu | 2006 | 110 | RFA | 37.0 | 34.0 | 57.3 | 58.0 | NR | NR | NR | NR | NR | NR | NR | NR | 38.0 | 37.3 | 64.1 | 63.1 |
| Luik | 2015 | 315 | CBA, RFA | 124.0 | 39.4 | 61.0 | 60.0 | NR | NR | 9.0 | 10.7 | 62.3 | 66.0 | 7.7 | 7.5 | NR | NR | NR | NR |
| Malmborg | 2013 | 110 | CBA, RFA | 27.0 | 24.5 | 59.0 | 62.0 | 28.0 | 27.0 | NR | NR | 41.0 | 63.0 | NR | NR | 40.0 | 42.0 | NR | NR |
| Mamchur | 2022 | 230 | CBA, RFA | 116.0 | 50.4 | 57.0 | 56.5 | 24.7 | 25.0 | NR | NR | 70.5 | 74.1 | 8.2 | 7.4 | NR | NR | NR | NR |
| Marrouche | 2022 | 843 | RFA | 178.0 | 21.1 | 62.2 | 63.2 | NR | NR | 9.5 | 10.7 | 58.7 | 58.5 | 8.6 | 8.1 | NR | NR | NR | NR |
| Marrouche | 2018 | 363 | RFA | 52.0 | 14.3 | 64.0 | 64 (median) | 29.0 | 29.1 | 24.0 | 36.0 | 72.0 | 74.0 | 12.0 | 11.0 | 48.0 | 49.5 | 32.5 | 31.5 |
| Masuda | 2025 | 342 | RFA | 167.0 | 50.0 | 73.8 | 74.7 | 23.7 | 23.7 | 25.0 | 21.0 | 68.0 | 74.0 | 9.0 | 11.0 | 44.1 | 43.6 | 55.8 | 57.4 |
| McLellan | 2015 | 234 | RFA | 79.0 | 34.0 | 59.1 | 59.0 | NR | NR | 3.0 | 5.0 | 38.0 | 38.0 | NR | NR | NR | NR | 59.8 | 59.2 |
| Mililis | 2023 | 199 | RFA, CBA | 37.0 | 18.6 | 60.2 | 62.7 | 30.1 | 33.1 | 15.0 | 13.6 | NR | NR | NR | NR | 44.9 | 43.6 | 55.2 | 55.7 |
| Miyamoto | 2025 | 499 | RFA, CBA | 136.0 | 27.0 | NR | NR | 24.4 | 24.3 | 16.1 | 14.0 | 63.1 | 64.4 | 5.2 | 7.6 | 41.5 | 42.0 | 60.0 | 60.0 |
| Mohanty | 2013 | 360 | RFA | 87.0 | 24.0 | 61.0 | 62.0 | 29.0 | 28.0 | 12.0 | 12.0 | 42.0 | 47.0 | 4.0 | 6.0 | 42.6 | 42.1 | 59.0 | 58.0 |
| Mont | 2014 | 146 | RFA | 33.0 | 22.6 | 55.0 | 55.0 | NR | NR | NR | NR | 46.9 | 39.5 | 100.0 | 2.1 | 41.3 | 42.7 | 61.1 | 60.8 |
| Morillo | 2014 | 127 | RFA | 31.0 | 24.4 | 56.3 | 54.3 | NR | NR | 1.5 | 6.6 | 42.4 | 41.0 | 4.6 | 6.6 | 40.0 | 43.0 | 61.4 | 60.8 |
| Nielsen | 2012 | 294 | RFA | 88.0 | 29.9 | 56.0 | 54.0 | 27.0 | 27.0 | 4.0 | 7.0 | 29.0 | 36.0 | 4.0 | 3.0 | 40.0 | 40.0 | NR | NR |
| Nilsson | 2006 | 100 | RFA | 29.0 | 29.0 | 57.0 | 55.0 | NR | NR | NR | NR | 22.0 | 20.0 | 7.0 | 13.0 | NR | NR | NR | NR |
| Oral | 2006 | 146 | RFA | NR | NR | NR | NR | NR | NR | NR | NR | NR | NR | NR | NR | NR | NR | NR | NR |
| Osmancik | 2025 | 203 | RFA, PFA | 64.0 | 31.5 | 59.8 | 60.0 | 35.0 | 34.9 | 19.0 | 29.1 | 84.0 | 83.5 | 3.0 | 2.9 | 45.0 | 45.6 | 59.2 | 61.2 |
| Packer | 2019 | 2204 | RFA | 819.0 | 37.2 | 68.0 | 67.0 | 30.0 | 30.0 | 25.3 | 25.7 | 79.1 | 82.2 | 10.6 | 9.4 | NR | NR | NR | NR |
| Packer | 2013 | 245 | CBA | 98.0 | 40.0 | 55.7 | 56.1 | 30.0 | 30.5 | 8.0 | 10.0 | 49.0 | 52.0 | 100.0 | 100.0 | 40.0 | 40.3 | 62.1 | 61.3 |
| Pak | 2021 | 314 | CBA, RFA | 90.0 | 28.7 | 60.8 | 59.0 | NR | NR | 11.5 | 10.8 | 51.3 | 44.9 | 7.7 | 10.8 | 38.8 | 39.6 | 65.2 | 65.9 |
| Pak | 2020 | 114 | RFA | 32.0 | 28.0 | 58.6 | 61.6 | NR | NR | 14.0 | 26.0 | 42.0 | 60.0 | NR | NR | 41.4 | 42.7 | 59.1 | 61.9 |
| Pappone | 2006 | 198 | RFA | 65.0 | 32.8 | 55.0 | 57.0 | NR | NR | 5.1 | 4.0 | 56.0 | 57.0 | NR | NR | 40.0 | 38.0 | 60.0 | 61.0 |
| Parkash | 2022 | 411 | RFA | 106.0 | 25.8 | 65.9 | 67.5 | 30.1 | 30.7 | 28.5 | 32.5 | 65.4 | 67.0 | 8.9 | 10.2 | 46.1 | 46.8 | NR | NR |
| Pavlovic | 2021 | 218 | CBA | 70.0 | 32.1 | 50.5 | 54.1 | NR | NR | 90.0 | 3.6 | 30.8 | 36.0 | NR | NR | 46.8 | 47.7 | 62.8 | 63.7 |
| Reddy | 2024 | 607 | PFA | NR | NR | 62.4 | 62.5 | 28.3 | 29.0 | 10.6 | 10.8 | 52.0 | 57.0 | 3.9 | 5.0 | 38.8 | 39.6 | 60.0 | 60.0 |
| Reynolds | 2010 | 159 | RFA | 53.0 | 33.0 | 55.5 | 55.8 | NR | NR | 10.0 | 13.0 | 49.0 | 50.0 | NR | NR | NR | NR | NR | NR |
| Rillig | 2017 | 258 | RFA | 83.0 | 32.2 | 64.1 | 62.4 | 27.3 | 27.7 | 7.0 | 6.0 | 60.0 | 58.0 | NR | NR | 42.0 | 42.1 | NR | NR |
| Sang | 2025 | 498 | RFA | 134.0 | 27.0 | 61.3 | 61.0 | NR | NR | 13.0 | 10.8 | 55.3 | 52.2 | 5.7 | 6.8 | 42.8 | 42.8 | 61.3 | 61.1 |
| Schmidt | 2017 | 134 | LBA, RFA | 49.0 | 36.6 | 65.0 | 66.0 | 28.0 | 30.0 | 9.0 | 11.0 | 71.0 | 74.0 | 3.0 | 3.0 | 43.0 | 43.0 | 61.0 | 61.0 |
| Sheikh | 2006 | 100 | RFA | 37.0 | 37.0 | NR | NR | NR | NR | NR | NR | NR | NR | NR | NR | NR | NR | NR | NR |
| Shen | 2025 | 302 | RFA | 137.0 | 45.0 | 63.0 | 65.9 | 24.6 | 24.7 | 10.0 | 8.0 | 27.0 | 25.0 | 7.0 | 5.0 | 41.0 | 40.0 | 61.0 | 60.3 |
| Shi | 2022 | 101 | RFA, CBA | 21.0 | 21.0 | 62.4 | 64.0 | 29.6 | 28.8 | 1.9 | 8.2 | 55.8 | 58.0 | NR | NR | 46.0 | 44.0 | 56.0 | 56.8 |
| Shim | 2017 | 108 | RFA | 25.0 | 23.1 | 59.7 | 61.9 | NR | NR | 17.0 | 20.0 | 52.8 | 56.4 | 28.3 | 29.1 | 46.1 | 44.0 | 57.8 | 60.7 |
| Shin | 2021 | 100 | RFA | 23.0 | 23.0 | 60.2 | 58.9 | 24.3 | 25.5 | 18.0 | 22.0 | 58.0 | 56.0 | 14.0 | 4.0 | 41.3 | 42.1 | 56.9 | 57.4 |
| Sohns | 2023 | 194 | RFA | 37.0 | 19.1 | 62.0 | 65.0 | 28.0 | 28.0 | 26.0 | 32.0 | NR | NR | NR | NR | 49.0 | 48.0 | 29.0 | 25.0 |
| Stabile | 2006 | 137 | RFA | 56.0 | 40.9 | 62.2 | 62.3 | NR | NR | NR | NR | 52.9 | 49.3 | 9.0 | 11.0 | 46.0 | 45.4 | 59.1 | 57.9 |
| Steven | 2013 | 102 | RFA | 33.0 | 32.0 | 62.8 | 62.4 | NR | NR | NR | NR | NR | NR | NR | NR | 37.0 | 39.8 | 62.8 | 62.2 |
| Strazdas | 2025 | 100 | CBA | 40.0 | 40.0 | 68.1 | 64.7 | 28.6 | 28.0 | 16.0 | 2.0 | 46.0 | 48.0 | NR | NR | 37.0 | 39.4 | 58.0 | 58.0 |
| Sultan | 2025 | 170 | RFA, CBA | 86.0 | 51.0 | 66.0 | 64.0 | 27.5 | 27.4 | 8.4 | 10.4 | 64.6 | 77.9 | 13.1 | 5.8 | 36.1 | 35.9 | 60.8 | 59.5 |
| Sun | 2022 | 390 | RFA | 161.0 | 41.3 | 63.1 | 61.6 | NR | NR | 16.0 | 14.0 | 40.0 | 40.0 | 5.0 | 7.0 | 37.3 | 37.2 | 63.1 | 63.3 |
| Takahashi | 2024 | 200 | RFA | 36.0 | 18.0 | 66.4 | 66.8 | 25.4 | 25.0 | 20.4 | 19.0 | NR | NR | 8.2 | 5.9 | 43.5 | 42.6 | 61.8 | 59.1 |
| Tamborero | 2010 | 146 | RFA | 24.0 | 17.0 | 51.9 | 53.2 | NR | NR | NR | NR | 41.4 | 53.4 | NR | NR | 43.6 | 42.6 | 55.6 | 59.1 |
| Tamborero | 2009 | 120 | RFA | 28.0 | 23.0 | 52.9 | 52.5 | NR | NR | NR | NR | 48.0 | 43.0 | NR | NR | 41.6 | 41.1 | 59.5 | 59.8 |
| Theis | 2022 | 150 | CBA, RFA | 64.0 | 43.0 | 61.6 | 66.1 | 27.2 | 27.2 | NR | NR | 59.0 | 73.0 | 5.3 | 9.3 | 39.0 | 41.0 | 55.7 | 56.4 |
| Theis | 2015 | 152 | RFA | 74.0 | 48.7 | 64.0 | 63.0 | 27.4 | 27.1 | NR | NR | 70.0 | 61.0 | NR | NR | 23.2 | 22.2 | 55.0 | 54.7 |
| Turco | 2007 | 107 | RFA | 38.0 | 36.0 | 57.0 | 57.0 | NR | NR | NR | NR | 61.0 | 57.0 | NR | NR | 48.0 | 48.0 | 57.0 | 57.0 |
| Ullah | 2016 | 117 | RFA | 50.0 | 42.7 | 59.0 | 59.0 | NR | NR | 2.0 | 9.0 | 24.0 | 38.0 | 3.0 | 9.0 | 41.0 | 38.0 | NR | NR |
| Ullah | 2014 | 166 | RFA | 34.0 | 22.0 | 60.0 | 60.0 | NR | NR | 8.0 | 8.0 | 10.0 | 13.0 | 8.0 | 8.0 | 42.0 | 41.0 | NR | NR |
| Wang | 2021 | 203 | RFA | 73.0 | 36.0 | 59.0 | 60.0 | 24.3 | 24.7 | 16.0 | 18.0 | 48.0 | 57.0 | 14.0 | 20.0 | 36.8 | 36.9 | 65.2 | 65.8 |
| Wang | 2008 | 106 | RFA | 48.0 | 45.0 | 66.6 | 65.4 | NR | NR | 1.9 | 0.0 | 19.0 | 23.0 | NR | NR | 36.9 | 36.5 | 62.1 | 62.4 |
| Wazni | 2020 | 203 | CBA | NR | NR | 60.4 | 61.4 | NR | NR | 14.0 | 17.0 | 56.0 | 58.0 | 2.0 | 3.0 | 38.7 | 38.2 | 60.9 | 61.1 |
| Wilber | 2010 | 167 | RFA | 56.0 | 33.5 | 55.5 | 56.1 | NR | NR | 9.5 | 12.0 | 48.6 | 50.0 | 1.9 | 5.0 | 40.0 | 40.5 | 62.3 | 62.7 |
| Wong | 2015 | 130 | RFA | 32.0 | 25.0 | 61.0 | 61.0 | 30.2 | 31.6 | NR | NR | NR | NR | NR | NR | 45.0 | 46.0 | NR | NR |
| Wu | 2021 | 648 | RFA | 227.0 | 35.0 | 64.8 | 64.8 | NR | NR | 26.3 | 23.7 | 61.5 | 63.2 | 12.5 | 13.1 | 45.0 | 46.0 | 53.3 | 51.9 |
| Wynn | 2016 | 122 | RFA | 42.0 | 32.0 | 41.0 | 47.0 | 28.6 | 29.2 | 3.0 | 9.0 | 64.0 | 52.0 | NR | NR | 43.0 | 43.0 | 61.5 | 60.5 |
| Xu | 2020 | 180 | RFA | 66.0 | 36.7 | 59.2 | 58.7 | 25.3 | 24.5 | 13.3 | 8.3 | 46.7 | 45.0 | 2.5 | 1.7 | 36.5 | 36.5 | 65.1 | 64.8 |
| Xu | 2012 | 123 | RFA | 43.0 | 35.0 | 61.5 | 60.9 | NR | NR | 12.1 | 22.8 | 40.9 | 35.1 | 7.6 | 10.5 | 39.2 | 39.1 | 58.5 | 58.1 |
| Yamaji | 2020 | 111 | RFA | 18.0 | 16.0 | 65.0 | 66.0 | 24.0 | 25.9 | NR | NR | NR | NR | NR | NR | 45.0 | 45.0 | 60.0 | 60.0 |
| Yang | 2022 | 300 | RFA | 89.0 | 32.0 | 60.6 | 60.4 | 27.8 | 25.8 | 7.8 | 10.5 | 48.5 | 40.8 | 3.7 | 2.8 | 41.4 | 42.5 | 61.3 | 62.1 |
| Yang | 2017 | 229 | RFA | 52.0 | 23.0 | 57.1 | 57.6 | 26.1 | 25.7 | NR | NR | NR | NR | NR | NR | 41.1 | 40.7 | 61.8 | 62.0 |
| Yao | 2020 | 214 | RFA | 38.0 | 18.0 | 56.6 | 58.0 | 27.3 | 26.3 | 18.0 | 18.0 | 51.0 | 47.0 | 8.0 | 11.0 | 41.8 | 42.6 | 60.7 | 60.5 |
| Yu | 2017 | 113 | RFA | 28.0 | 25.0 | 61.4 | 59.3 | 24.9 | 25.1 | 20.0 | 13.0 | 54.0 | 52.0 | 25.0 | 19.0 | 42.6 | 42.7 | 61.2 | 62.9 |
| Zhang | 2017 | 342 | RFA | 117.0 | 34.0 | 58.0 | 58.0 | NR | NR | 8.2 | 6.0 | 57.1 | 56.8 | 8.8 | 7.2 | 38.7 | 40.1 | 55.8 | 58.6 |

**Table S3. Arrhythmia Endpoint Definitions and Composite vs Individual Endpoints**

| **First Author** | **Year Published** | **Primary end point definition for tachyarrhythmia** | **Main AF Endpoint Measure** | **Composite or Individual Endpoint** | **Treatment Arm Results (AF Recurrence %)** | **Control Arm Results (AF Recurrence %)** | **AF End point Follow up time (months)** |
| --- | --- | --- | --- | --- | --- | --- | --- |
| Ahn | 2023 | AF/AT/AFL ≥30 s | Recurrence of AF/AT/AFL >30 s after blanking | Individual | 16.0 | 18.0 | 12.0 |
| Ahn | 2022 | AF/AT/AFL ≥30 s | Recurrence of AF/AT/AFL >30 s after blanking | Individual | 24.0 | 46.0 | 15.0 |
| Al-Kaisey | 2023 | AF/AT/AFL ≥30 s | Recurrence of AF/AT/AFL >30 s after blanking & AF Burden reduction | Composite | 47.0 | 96.0 | 12.0 |
| Andrade | 2021 | AF/AT/AFL ≥30 s | Recurrence of AF/AT/AFL ≥30 s after 90-day blanking (continuous ILR) | Individual | 43.0 | 68.0 | 12.0 |
| Arbelo | 2014 | AF/AT/AFL ≥30 s | Freedom from any AF/AT/AFL ≥30 s | Individual | 41.0 | 44.0 | 15.0 |
| Arentz | 2007 | AF/AT ≥30 s | Freedom from AF/AT >30 s, off AADs, symptom free after single procedure | Composite | 51.0 | 33.0 | 15.0 |
| Ariyaratnam | 2025 | AF/AT/AFL ≥30 s | Freedom from AF/AT/AFL >30 s, off AAD after single procedure (2-year primary) | Composite | 62.5 | 63.4 | 24.0 |
| Aryana | 2021 | AF/AT/AFL ≥30 s | Recurrence of AF/AT/AFL >30 s after blanking (single procedure) | Individual | 25.5 | 45.5 | 12.0 |
| Baek | 2021 | AF/AT ≥30 s | Freedom from AF/AT ≥30 s after single procedure | Individual | 25.3 | 42.0 | 16.0 |
| Baimbetov | 2022 | AF/AT/AFL ≥30 s | Freedom from AF/AT/AFL >30 s and no AAD use or repeat ablation | Composite | 30.0 | 28.0 | 36.0 |
| Blomström-Lundqvist | 2019 | NR | Freedom from AF ≥2 min | Individual | 25.3 | 29.7 | 12.0 |
| Brachmann | 2025 | AF/AT/AFL ≥30 s | Freedom from AF/AT >30 s after single procedure (3–12 months) | Composite | 30.7 | 32.5 | 12.0 |
| Buist | 2018 | AF/AT/AFL ≥30 s | Freedom from any atrial arrhythmia (>30 s) off AAD after blanking | Composite | 25.0 | 42.0 | 12.0 |
| Bänsch | 2013 | AF/AT/AFL ≥30 s | Freedom from any atrial arrhythmia >30 s with no blanking after single procedure | Individual | 48.0 | 45.0 | 10.0 |
| Cao | 2025 | AF/AT/AFL ≥30 s | Freedom from AF/AT/AFL ≥30 s off AAD after single procedure | Individual | 21.0 | 26.6 | 12.0 |
| Caponi | 2010 | AF/AT/AFL ≥30 s | Maintenance of sinus rhythm off AAD | Individual | 66.0 | 68.0 | 12.0 |
| Castro-Urda | 2025 | AF/AT/AFL ≥30 s | Freedom from AF/AT/AFL >30 s after single procedure | Individual | 37.1 | 28.0 | 12.0 |
| Chen | 2023 | AF/AT/AFL ≥30 s | Freedom from AF/AT/AFL ≥30 s after single ablation | Individual | 15.0 | 24.0 | 23.0 |
| Choi | 2022 | AF/AT ≥30 s | Freedom from AF/AT/AFL >30 s after single procedure | Individual | 26.0 | 24.0 | 12.0 |
| Chun | 2021 | AF/AT ≥30 s | Freedom from AF/AT/AFL >30 s and no AAD use | Composite | 80.0 | 78.0 | 12.0 |
| Corrado | 2010 | AF ≥30 s | Sinus rhythm maintenance and no AAD use | Composite | 26.0 | 19.0 | 12.0 |
| Darkner | 2014 | AF/AT/AFL ≥30 s | Recurrence of AF/AT/AFL >30 s | Individual | 39.0 | 48.0 | 6.0 |
| Deisenhofer | 2025 | AF ≥30 s | Freedom from AF/AT/AFL >30 s and no repeat ablations | Composite | 12.0 | 30.0 | 12.0 |
| DiBiase | 2016 | AF/AT/AFL ≥30 s | Freedom from AF/AT/AFL >30 s and no AAD use | Composite | 30.0 | 66.0 | Unclear |
| Ding | 2022 | AF/AT/AFL ≥30 s | Occurence of persistent AF/AT or an AAD | Composite | 41.9 | 72.0 | 36.0 |
| Dixit | 2008 | AF/AT/AFL ≥2 min | Long-term AF control (freedom or ≥90% burden reduction) after single procedure; ≥2 min threshold; 6-week blanking | Composite | 25.0 | 29.0 | 12.0 |
| Dong | 2015 | AF/AT/AFL ≥30 s | Freedom from AF/AT/AFL >30 s off AAD after blanking (single procedure) | Composite | 33.0 | 40.0 | 12.0 |
| Dukkipati | 2015 | AF ≥1 min | Freedom from AF ≥1 min, any AFL/AT and no AAD use, PV isolation failure, or redo ablation | Composite | 20.4 | 24.0 | 12.0 |
| Dulai | 2024 | AF/AT/AFL ≥30 s | AF burden reduction | Individual | NR | NR | 6.0 |
| Duytschaever | 2018 | AF/AT/AFL ≥30 s | Recurrence of AF/AT/AFL >30 s | Individual | 2.7 | 21.9 | 12.0 |
| Ejima | 2019 | AF/AT/AFL ≥30 s | Freedom from AF/AT/AFL >30 s off AADs after single procedure | Composite | 15.0 | 30.0 | 12.0 |
| Estner | 2011 | AF/AT/AFL ≥30 s | Freedom from atrial tachyarrhythmia, off AADs after single procedure | Composite | 61.0 | 63.0 | 12.0 |
| Falasconi | 2025 | AF/AT/AFL ≥30 s | Freedom from AF/AT/AFL >30 s after blanking (single procedure) | Individual | 18.7 | 22.4 | 12.0 |
| Fassini | 2005 | AF/AT/AFL ≥30 s | Freedom from AF/AT/AFL >30 s at 12 months | Individual | 29.0 | 47.0 | 12.0 |
| Faustino | 2015 | AF/AT ≥30 s | Recurrence of AF/AT >30 s | Individual | 26.7 | 46.7 | 12.0 |
| Ferrero-de-Loma-Osorio | 2017 | AF ≥30 s | Freedom from AF/AT/AFL ≥30 s, off AAD | Individual | 22.0 | 21.0 | 12.0 |
| Fiala | 2008 | AF/AT/AFL ≥30 s | Freedom from AF/AT/AFL >30 s, off AADs after single procedure | Composite | 43.0 | 44.0 | 48.0 |
| Fichtner | 2013 | NR | Freedom from AF (7-day Holter) off AAD after single procedure | Individual | 47.0 | 41.0 | 12.0 |
| Fink | 2017 | AF/AT/AFL ≥30 s | Freedom from AF/AT/AFL >30 s | Individual | 42.0 | 45.0 | 12.0 |
| Gaita | 2008 | AF/AFL ≥30 s | Sinus rhythm maintenance, off AADs | Composite | 18.9 | 46.9 | 36.0 |
| Gal | 2014 | AF ≥30 s | Freedom from AF/AT/AFL | Individual | 55.0 | 52.0 | 60.0 |
| Gallagher | 2021 | AF/AT ≥30 s | Sinus rhythm maintenance | Individual | 67.0 | 76.0 | 60.0 |
| Gula | 2016 | AF/AT/AFL ≥30 s | Freedom from AF ≥30 s (single procedure) | Individual | 35.6 | 41.7 | 12.0 |
| Inoue | 2021 | AF/AT/AFL ≥30 s | Freedom from AF/AT/AFL >30 | Individual | 19.7 | 29.5 | 12.0 |
| Jaïs | 2008 | AF ≥3 min | Freedom from AF ≥3 min and no AAD use or crossover | Composite | 23.0 | 89.0 | 12.0 |
| Jiang | 2022 | AF/AT/AFL ≥30 s | Freedom from AF/AT/AFL >30 s, off AADs | Composite | 29.0 | 37.0 | 12.0 |
| Kaiser | 2024 | AF/AT/AFL ≥30 s | Freedom from AF/AT/AFL >30 s | Individual | 32.0 | 44.0 | 12.0 |
| Kalman | 2023 | AF/AT ≥30 s | Freedom from atrial arrhythmia >30 sec post-ablation | Individual | 43.7 | 41.4 | 12.0 |
| Kang | 2014 | AF/AT ≥30 s | Recurrence AF/AT ≥30s | Composite | 6.0 | 27.0 | 12.0 |
| Karch | 2005 | AF/AT/AFL ≥30 s | Freedom from AT ≥30 s | Individual | 58.0 | 44.0 | 6.0 |
| Kim | 2025 | AF/AT/AFL ≥30 s | Freedom from AF/AT/AFL ≥30 s | Individual | 37.5 | 53.1 | 24.0 |
| Kim | 2010 | AF/AT/AFL ≥30 s | Maintenance of sinus rhythm | Individual | 20.4 | 18.9 | 23.0 |
| Kim | 2024 | AF/AT/AFL ≥30 s | Freedom from AF/AT/AFL ≥30 s without AAD use | composite | 29.2 | 45.6 | 24.0 |
| Kircher | 2018 | AF/AT/AFL ≥30 s | Freedom from AF/AT/AFL >30 s off AAD after single procedure | Composite | 32.0 | 58.0 | 12.0 |
| Kisler | 2023 | AF/AT/AFL ≥30 s | Freedom from AF/AT/AFL >30 s and no AAD use | Composite | 47.6 | 46.4 | 12.0 |
| Kobori | 2015 | AT ≥30 s | recurrent atrial tachyarrhythmias lasting for >30 s or those requiring repeat ablation, hospital admission, or AADs | Composite | 31.3 | 32.9 | 12.0 |
| Kuck | 2016 | AF/AT/AFL ≥30 s | Recurrence of AF >30 s, or any AFL/AT, or need for other treatment | Composite | 35.9 | 34.6 | 12.0 |
| Kuck | 2021 | AT ≥30 s | Progression to persistent AF/AT | Individual | 49.2 | 84.8 | 36.0 |
| Kuck | 2019 | NR | AF burden reduction | Individual | 26.5 | 50.0 | 12.0 |
| Kuniss | 2021 | AF/AT/AFL ≥30 s | Freedom from AF/AT/AFL >30 s | Individual | 17.8 | 32.6 | 12.0 |
| Lee | 2018 | AF/AT/AFL ≥30 s | Recurrence of AF/AT/AFL >30 s after 90-day blanking | Individual | 20.2 | 25.8 | 12.0 |
| Lee | 2019 | AF/AT ≥30 s | Freedom from AF/AT recurrence >30 s, off AADs | Composite | 67.1 | 68.9 | 22.0 |
| Lee | 2019 | AF/AT ≥30 s | Recurrence of AF/AT ≥30 s | Individual | 23.8 | 26.5 | 16.0 |
| Lin | 2023 | AF/AT/AFL ≥30 s | Freedom from AF/AT/AFL >30 s | Individual | 32.9 | 57.6 | 12.0 |
| Lin | 2019 | AF ≥30 s | Recurrence of AF >30 s | Individual | 2.8 | 14.1 | 7.0 |
| Lin | 2012 | AF ≥1 min | Freedom from symptomatic atrial arrhythmia | Individual | 16.4 | 29.1 | 16.0 |
| Liu | 2006 | AT ≥10 min | Freedom from Ata, off AADs | Composite | 16.4 | 21.8 | 9.0 |
| Luik | 2015 | AF/AT ≥30 s | Freedom from AF >30 s and no complications | Composite | 35.9 | 36.9 | 6.0 |
| Malmborg | 2013 | AF ≥30 s | Freedom from symptomatic AF >30 s and no AAD use | Composite | 54.0 | 66.0 | 12.0 |
| Mamchur | 2022 | AF/AT/AFL ≥30 s | Freedom from atrial tachycardia recurrence | Individual | 21.2 | 16.7 | 12.0 |
| Marrouche | 2022 | AF/AT/AFL ≥30 s | Recurrence of AF/AT/AFL or repeat ablation | Composite | 43.0 | 46.1 | 12.0 |
| Marrouche | 2018 | AT ≥30 s | AF burden reduction | Individual | 37.0 | 78.0 | 60.0 |
| Masuda | 2025 | AF/AT ≥30 s | Freedom from AF/AT ≥30 s off AADs | Composite | 39.0 | 50.0 | 12.0 |
| McLellan | 2015 | AF/AT ≥30 s | Freedom from AF/AT >30 s and no repeat ablations | Composite | 38.0 | 30.0 | 17.0 |
| Mililis | 2023 | AF/AT/AFL ≥30 s | Recurrence of AF/AT/AFL >30 s | Individual | 26.3 | 27.0 | 12.0 |
| Miyamoto | 2025 | AF/AT/AFL ≥30 s | Freedom from AF/AT/AFL ≥30 s after 90-day blanking (single procedure) | Individual | 22.5 | 23.2 | 12.0 |
| Mohanty | 2013 | AF/AT/AFL ≥30 s | Freedom from AF/AT/AFL >30 s off AAD after blanking | Composite | 36.0 | 81.0 | 21.0 |
| Mont | 2014 | AF/AT ≥30 s | Freedom from sustained AF/AT >24 h or requiring cardioversion | Composite | 29.6 | 56.8 | 12.0 |
| Morillo | 2014 | AF/AT/AFL ≥30 s | Recurrence of AF/AT/AFL >30 s | Individual | 54.5 | 72.1 | 24.0 |
| Nielsen | 2012 | AF ≥1 min | Freedom from any AF | Individual | 15.0 | 29.0 | 24.0 |
| Nilsson | 2006 | AF ≥10 min | Freedom from symptomatic AF/AT ≥10 min, off AAD after final ablation | Composite | 43.0 | 69.0 | 12.0 |
| Oral | 2006 | AF/AT/AFL ≥3 s | Freedom from AF/AFL, off AADs | Composite | 26.0 | 42.0 | 12.0 |
| Osmancik | 2025 | AF/AT/AFL ≥30 s | Freedom from AF/AT/AFL ≥30 s | Individual | 27.0 | 65.4 | 12.0 |
| Packer | 2019 | AF ≥30 s | Recurrence of AF ≥30 s | Individual | 36.4 | 59.2 | 60.0 |
| Packer | 2013 | AF ≥30 s | Freedom from AF ≥30 s and no AAD use or redo ablation | Composite | 30.1 | 92.7 | 12.0 |
| Pak | 2021 | AF/AT ≥30 s | Freedom from AF/AT >30 s and no AAD use | Composite | 29.5 | 26.6 | 9.8 |
| Pak | 2020 | AF/AT ≥30 s | Recurrence of AF/AT ≥30 s | Individual | 28.0 | 32.0 | 24.0 |
| Pappone | 2006 | AT ≥30 s | Freedom from atrial tachyarrhythmia ≥30 s | Individual | 86.0 | 22.0 | 12.0 |
| Parkash | 2022 | NR | Sinus rhythm maintenance | Individual | 85.6 | 12.9 | 24.0 |
| Pavlovic | 2021 | AF/AT/AFL ≥30 s | Recurrence from AF/AT/AFL >30s | Individual | 17.8 | 32.4 | 12.0 |
| Reddy | 2024 | AT ≥30 s | Recurrence of AT ≥30 s | Individual | 17.2 | 16.4 | 12.0 |
| Reynolds | 2010 | NR | Freedom from recurrent symptomatic AF/AT/AFL | Individual | 30.0 | 19.0 | 9.0 |
| Rillig | 2017 | AF/AT/AFL ≥30 s | Freedom from AF/AT recurrence | Individual | 23.6 | 20.2 | 12.0 |
| Sang | 2025 | AF/AT/AFL ≥30 s | Freedom from AF/AT/AFL >30 s off AAD | Composite | 29.3 | 38.5 | 12.0 |
| Schmidt | 2017 | AT ≥30 s | Freedom from AF/AT/AFL >30 s | Individual | 28.8 | 30.7 | 12.0 |
| Sheikh | 2006 | NR | Freedom from AF after single ablation (on or off AADs) | Individual | 18.0 | 10.0 | 9.0 |
| Shen | 2025 | AF/AT/AFL ≥30 s | Freedom from AF/AT/AFL >30 s off AAD | Composite | 13.2 | 19.2 | 12.0 |
| Shi | 2022 | AF/AT/AFL ≥30 s | Freedom from AF/AT/AFL >30 s | Individual | 27.5 | 38.0 | 12.0 |
| Shim | 2017 | AF/AT/AFL ≥30 s | Recurrence of AF/AT/AFL ≥30 s | Individual | 14.0 | 18.9 | 12.0 |
| Shin | 2021 | AF/AT/AFL ≥30 s | Recurrence of AF/AT/AFL >30 s | Individual | 16.3 | 24.5 | 12.0 |
| Sohns | 2023 | AF ≥30 s | AF burden reduction | Individual | NR | NR | 12.0 |
| Stabile | 2006 | AF/AT ≥30 s | Freedom from AF/AT | Individual | 44.1 | 91.3 | 12.0 |
| Steven | 2013 | AF/AT ≥30 s | Freedom from AF/AT >30 s off AAD | Composite | 17.0 | 48.0 | 12.0 |
| Strazdas | 2025 | AF/AT/AFL ≥30 s | Freedom from AF/AFL/AT ≥30 s off AAD | Composite | 24.0 | 12.0 | 12.0 |
| Sultan | 2025 | NR | Event-free survival (no AF/AT/AFL, no AAD, no re-ablation) | Composite | 25.0 | 18.6 | 12.0 |
| Sun | 2022 | AF/AT/AFL ≥30 s | Freedom from AF/AT recurrence | Individual | 18.8 | 26.4 | 12.0 |
| Takahashi | 2024 | AF/AT ≥30 s | Freedom from AF/AT/AFL >30 s off AAD | Composite | 32.7 | 27.5 | 24.0 |
| Tamborero | 2010 | AF/AT/AFL ≥30 s | Freedom from AF/AT/AFL ≥30 s off AAD | Composite | 35.6 | 57.5 | 9.0 |
| Tamborero | 2009 | AF/AT/AFL ≥30 s | Freedom from AF/AT/AFL >30 s | Individual | 38.0 | 40.0 | 10.0 |
| Theis | 2022 | AF/AT/AFL ≥30 s | Freedom from any atrial arrhythmia >30 s after single procedure off AADs | Composite | 14.7 | 13.3 | 12.0 |
| Theis | 2015 | AF/AT/AFL ≥30 s | Freedom from AF/AT/AFL >30 s | Individual | 13.0 | 32.0 | 12.0 |
| Turco | 2007 | AF/AT/AFL ≥30 s | Freedom from AF/AT/AFL >30 s | Individual | 30.0 | 34.0 | 12.0 |
| Ullah | 2016 | AF/AT ≥30 s | Freedom from AF/AT ≥30 s off AAD | Composite | 51.0 | 48.0 | 12.0 |
| Ullah | 2014 | AF/AT ≥30 s | Freedom from AF/AT ≥30 s off AAD after single procedure | Composite | 76.0 | 67.0 | 12.0 |
| Wang | 2021 | AF/AT/AFL ≥1 min | Freedom from AF/AT/AFL >1 min | Individual | 14.7 | 26.7 | 12.0 |
| Wang | 2008 | AF/AT/AFL ≥30 s | Freedom from AF/AT/AFL >30 s | Individual | 7.4 | 5.8 | 12.0 |
| Wazni | 2020 | AT ≥30 s | Freedom from AF and no AAD use | Composite | 25.4 | 55.0 | 12.0 |
| Wilber | 2010 | AF ≥15 s | Freedom from AF >15 s and no AAD use or late reablation | Composite | 34.0 | 84.0 | 12.0 |
| Wong | 2015 | AF/AT ≥30 s | Freedom from AF/AT at 12 months off AAD | Composite | 54.0 | 43.0 | 12.0 |
| Wu | 2021 | AF/AT/AFL ≥30 s | Recurrence of AF | Individual | 39.4 | 79.1 | 60.0 |
| Wynn | 2016 | AF/AT ≥30 s | Freedom from AF/AT ≥30 s | Individual | 38.0 | 32.0 | 12.0 |
| Xu | 2020 | AF/AT/AFL ≥30 s | Freedom from atrial tachyarrhythmia >30 s after single procedure | Individual | 15.0 | 20.3 | 18.0 |
| Xu | 2012 | AF ≥3 min | Freedom from AF ≥3 min | Individual | 37.9 | 77.2 | 6.0 |
| Yamaji | 2020 | AF/AT ≥30 s | Freedom from AF/AT >30 s | Individual | 19.0 | 41.0 | 15.0 |
| Yang | 2022 | AF/AT/AFL ≥30 s | Freedom from AF/AT/AFL ≥30 s off AADs | Composite | 32.8 | 32.6 | 18.0 |
| Yang | 2017 | AF/AT ≥30 s | Freedom from AF/AT >30 s off AADs | Composite | 26.0 | 28.5 | 18.0 |
| Yao | 2020 | AF/AT ≥30 s | Freedom from AF/AT >30 s | Individual | 40.0 | 43.0 | 24.0 |
| Yu | 2017 | AF/AT ≥30 s | Freedom from AF/AT ≥30 s | Individual | 19.0 | 30.0 | 19.0 |
| Zhang | 2017 | AF/AT/AFL ≥30 s | Freedom from AF/AFL/AT >30s off AAD | Composite | 32.4 | 31.1 | 12.0 |

**Table S4. Blanking Period and Monitoring Methods**

| **First Author** | **Year Published** | **Follow-up Appointments in Number of Months** | **Method of follow-up (Holter, ECG, loop recorder)** | **Blanking period (days)** | **Blanking period recurrence - reported (Yes/No)** |
| --- | --- | --- | --- | --- | --- |
| Ahn | 2023 | 1, 3, 6, 9, 12 | ECG, 72-h Holter | 90 | No |
| Ahn | 2022 | 1, 3, 6, 9, 12 & 6 monthly afterwards | ECG, 24-h Holter | 90 | No |
| Al-Kaisey | 2023 | 3, 6, 12 | AliveCor KardiaMobile ECG (twice daily), 24-h Holter, loop recorder | 90 | No |
| Andrade | 2021 | 3, 6, 12 | Implantable loop recorder | 90 | No |
| Arbelo | 2014 | 1, 3, 6, 12 & 6 monthly afterwards | 48‑h Holter at each visit + symptom‑driven ECG | 90 | Yes |
| Arentz | 2007 | 1, 3, 6, 15 | ECG, 24-hour Holter, event recorder | 30 | Yes |
| Ariyaratnam | 2025 | 1, 3, 6, 9, 12, 18, 24 | 7-day Holter | 90 | No |
| Aryana | 2021 | 3, 6, 12 | ECG, 7–14-day mobile telemetry | 90 | no |
| Baek | 2021 | 1, 3, 6, 12 & then 6 monthly as required | ECG, 24-hour Holter, event recorder | 90 | Yes |
| Baimbetov | 2022 | 3, 6, 9, 12, 18, 24, 36 | ILR/Implanted cardiac device monitoring, CT, Echocardiogram | 90 | No |
| Blomström-Lundqvist | 2019 | 3, 6, 9, 12, 18, 24, 30, 36, 42, 48 | ILR/Implanted cardiac device monitoring, Quality-of-life questionnaires, Symptom questionnaire/diary, 24-hour Holter, Echocardiogram | 90 | No |
| Brachmann | 2025 | 1, 3, 6, 12 | ECG, 24–72-h Holter or device interrogation | 90 | Yes |
| Buist | 2018 | 3, 6, 12 | ECG each visit + 24–72 h Holter at 6 & 12 months | 90 | No |
| Bänsch | 2013 | 3, 6, 9 & then 3 monthly | ECG, 4–7-day Holter | 0 | No |
| Cao | 2025 | 3, 6, 12 | ECG, 7-day Holter | 90 | Yes |
| Caponi | 2010 | 3, 6, 12 | ECG, 24-hr Holter | 90 | No |
| Castro-Urda | 2025 | 3, 6, 12 | AliveCor Kardia mobile ECG, ECG, Holter | 90 | No |
| Chen | 2023 | 3, 6, 12 & then 6 monthly | ECG, 24-hour Holter, 7-day Holter | 90 | No |
| Choi | 2022 | 1, 3, 6, 12 | ECG, 24-hour Holter | 90 | Yes |
| Chun | 2021 | 3, 6, 12 | 72-hour Holter, Clinic visits, Transtelephonic monitor/Event monitor | 90 | No |
| Corrado | 2010 | 1, 3, 6, 9, 12 | ECG, 48-hour Holter, event recorder, device interrogation | 56 | No |
| Darkner | 2014 | 1, 3, 6 | 72-hour Holter, Transtelephonic monitor/Event monitor | 90 | Yes |
| Deisenhofer | 2025 | 3, 6, 12 | Transtelephonic monitor/Event monitor, 24-hour Holter, Some ILR used but not universal | 90 | No |
| DiBiase | 2016 | 3, 6, 12, 24 | ILR/Implanted cardiac device monitoring, periodic ECG, Echocardiogram | 90 | Yes |
| Ding | 2022 | 1, 3, 6, 12, 24, 36 | ECG, 24-hour Holter, 7-day Holter | 90 | No |
| Dixit | 2008 | 1.5, 6, 12 | ECG, transtelephonic monitoring (3‑week periods) | 42 | Yes |
| Dong | 2015 | 3, 6, 12 | Transtelephonic ECG, 48-hour Holter, ECG | 90 | No |
| Dukkipati | 2015 | 1, 3, 6, 12 | Transtelephonic monitor/Event monitor, Holter of unspecified duration | 90 | Yes |
| Dulai | 2024 | 3, 6 | ILR/Implanted cardiac device monitoring | 90 | No |
| Duytschaever | 2018 | 3, 6, 12 | Periodic ECG, 24-hour Holter, 7-day Holter, Transtelephonic monitor/Event monitor | 90 | No |
| Ejima | 2019 | 1, 3, 6, 9, 12 | ECG, 24-hour Holter | 90 | No |
| Estner | 2011 | 1, 3, 6, 12 | ECG, 7-day Holter | 70 | No |
| Falasconi | 2025 | 1, 3, 6, 12 | ECG, 24-hour Holter | 90 | Yes |
| Fassini | 2005 | 1, 3, 6, 9, 12 | ECG (transtelephonic), 24-hour Holter | 60 | No |
| Faustino | 2015 | 3, 6, 12 | ECG, 48-hour Holter | 90 | No |
| Ferrero-de-Loma-Osorio | 2017 | 3, 6, 12 | ECG, 30-day Holter | 90 | No |
| Fiala | 2008 | 1.5, 3, 6, 9, 12 & then 6 monthly | ECG, 24-h Holter, transtelephonic ECG | 42 | No |
| Fichtner | 2013 | 3, 6, 12 | 7-day Holter ECG | NR | NR |
| Fink | 2017 | 3, 6, 12 | ECG, 24-hour Holter, telephone | 90 | No |
| Gaita | 2008 | 1, 3, 6, 12, 18, 24, 36 | ECG, 24-hour Holter, event recorder | 60 | No |
| Gal | 2014 | 3, 6, 12 & then as required | ECG, 24-hour Holter | 90 | No |
| Gallagher | 2021 | 6, 12, 36, 60 | 48-hour Holter, periodic ECG | 180 | No |
| Gula | 2016 | 3, 6, 12 | ECG, 24-hour Holter, 2-week event recorder | NR | Yes |
| Inoue | 2021 | 1, 3, 6, 9, 12 | ECG, 24-hour Holter, event monitor | 90 | Yes |
| Jaïs | 2008 | 3, 6, 12 | 24-hour Holter, periodic ECG, Symptom questionnaire/diary, Quality-of-life questionnaire, Exercise test | 90 | No |
| Jiang | 2022 | 3, 6, 9, 12 | ECG, 24-hour Holter, handheld ECG (weekly, symptom-triggered) | 90 | Yes |
| Kaiser | 2024 | 3, 6, 9, 12 | 48-hour Holter, ECG, event recorder | 90 | No |
| Kalman | 2023 | 3, 6, 12 | Transtelephonic monitor/Event monitor, 24-hour Holter, Some ILR used but not universal | 90 | Yes |
| Kang | 2014 | 1, 3, 6, 12 | ECG, 24/48-h Holter, event recorder | 90 | Yes |
| Karch | 2005 | 1, 3, 6 | 7-day Holter | 30 | No |
| Kim | 2025 | 1, 3, 6, 12, 18, 24 | ECG, 24–72-hour Holter, event recorder | 90 | No |
| Kim | 2010 | 3, 6, 12 & then 6 monthly | ECG, 24-hour Holter | 90 | Yes |
| Kim | 2024 | 1, 3, 6, 12, 18, 24 | ECG, 24–72 h Holter, event recorder | 90 | No |
| Kircher | 2018 | 6, 12 | ECG, 7-day Holter | 90 | No |
| Kisler | 2023 | 2, 3, 6, 12 | Transtelephonic monitor/Event monitor, 24-hour Holter, Some ILR used but not universal | 90 | No |
| Kobori | 2015 | 3, 6, 12 | ECG, 2-week ambulatory ECG, 24-h Holter | 90 | Yes |
| Kuck | 2016 | 3, 6, 12 | 24-hour Holter, periodic ECG, Transtelephonic monitor/Event monitor | 90 | No |
| Kuck | 2021 | 12, 24, 36 | Transtelephonic monitor/Event monitor, Symptom questionnaire/diary | 90 | No |
| Kuck | 2019 | 1, 3, 6, 12 | Transtelephonic monitor/Event monitor, ILR/Implanted cardiac device monitoring | 90 | No |
| Kuniss | 2021 | 1, 3, 6, 9, 12 | 7-day Holter, periodic ECG | 90 | Yes |
| Lee | 2018 | 1, 3, 6, 9, 12 & then 6 monthly | ECG, 24-hour Holter, 1-month event recorder | 90 | Yes |
| Lee | 2019 | 1, 3, 6, 9, 12 | ECG, 24-hour Holter, event recorder | 90 | Yes |
| Lee | 2019 | 1, 3, 6, 12 then 6 monthly | ECG, 24-h Holter, event recorder | 90 | Yes |
| Lin | 2023 | 6, 12 | ECG, 24h Holter | NR | No |
| Lin | 2019 | 1, 3, 6, 12 | ECG, 48-h Holter | 90 | No |
| Lin | 2012 | 3, 6, 9, 12 | ECG + 24-h Holter/7-day event monitors | 60 | Yes |
| Liu | 2006 | 1, 3, 6, 9 | ECG, 24-hour Holter, telephone | 90 | Yes |
| Luik | 2015 | 3, 6, 9, 12 | 24-hour Holter, 7-day Holter | 90 | No |
| Malmborg | 2013 | 3, 6, 12 | Periodic ECG, 7-day Holter | 90 | No |
| Mamchur | 2022 | 3, 6, 12 | Periodic ECG, 24-hour Holter | 90 | No |
| Marrouche | 2022 | 3, 6, 12 | Transtelephonic monitor/Event monitor | 90 | No |
| Marrouche | 2018 | 3, 6, 12, 24, 36, 48, 60 | ILR/Implanted cardiac device monitoring | 84 | No |
| Masuda | 2025 | 6, 12 | ECG, 24-hour Holter, portable ECG (twice-daily) | 90 | No |
| McLellan | 2015 | 1, 3, 6, 12, 17 | Transtelephonic monitor/Event monitor, 7-day Holter | 90 | No |
| Mililis | 2023 | 1, 3, 6, 9, 12 | 24-hour Holter, periodic ECG | 90 | Yes |
| Miyamoto | 2025 | 1, 3, 12 | ECG, 24-h Holter, ambulatory electrogram twice daily | 90 | No |
| Mohanty | 2013 | 3, 6, 9, 12 | ECG, 7-day Holter, event recorder | 84 | No |
| Mont | 2014 | 1, 3, 6, 12 | Periodic ECG, 24-hour Holter | 90 | Yes |
| Morillo | 2014 | 1, 3, 6, 12, 24 | Transtelephonic monitor/Event monitor | 90 | No |
| Nielsen | 2012 | 3, 6, 12, 18, 24 | 7-day Holter | 90 | No |
| Nilsson | 2006 | 1, 3, 6, 9, 12 | ECG, 14-day Holter, event recorder | 30 | No |
| Oral | 2006 | 3, 6, 12 | Transtelephonic ECG monitoring, clinic ECGs | 90 | No |
| Osmancik | 2025 | 1, 3, 6, 9, 12 | 7-day Holter, periodic ECG, Symptom questionnaire/diary | 90 | No |
| Packer | 2019 | 3, 6, 12, 18, 24, 30, 36, 42, 48, 54, 60 | Transtelephonic monitor/Event monitor, 96-hour Holter | 90 | No |
| Packer | 2013 | 1, 3, 6, 9, 12 | 24-hour Holter, periodic ECG, Transtelephonic monitor/Event monitor | 90 | No |
| Pak | 2021 | 1, 3, 6, 12 | Periodic ECG, 24-hour Holter | 90 | Yes |
| Pak | 2020 | 1, 3, 6, 12 & then 6 monthly as required | ECG, 24-hour Holter | 90 | Yes |
| Pappone | 2006 | 3, 6, 12 | 48-hour Holter, periodic ECG, echocardiogram, transtelephonic monitor/event monitor | 42 | No |
| Parkash | 2022 | 2, 4, 6, 12, 18, 24 | Periodic ECG, Exercise test, Quality-of-life questionnaire, Echocardiograms, 14-day CardioSTAT | 0 | No |
| Pavlovic | 2021 | 1, 3, 6, 9, 12 | Holter, Symptom questionnaire/diary, Quality-of-life questionnaire | 90 | No |
| Reddy | 2024 | 6, 12 | 72-hour Holter, Transtelephonic monitor/Event monitor | 90 | No |
| Reynolds | 2010 | 3, 6, 9 | ECG, transtelephonic monitoring | 90 | No |
| Rillig | 2017 | 3, 6, 12 | 72-hour Holter, Transtelephonic monitor/Event monitor | 90 | No |
| Sang | 2025 | 1, 3, 6, 12 | Wearable single-lead ECG patch (24 h/week), symptom-triggered ECG, Holter monitoring | 90 | No |
| Schmidt | 2017 | 3, 6, 12 | 72-hour Holter, Transtelephonic monitor/Event monitor | 90 | No |
| Sheikh | 2006 | NR | NR | 90 | No |
| Shen | 2025 | 3, 6, 12, 18, 24 | ECG, 24-hour Holter (3,6 months), 7-day Holter (12 months) | 90 | No |
| Shi | 2022 | 3, 6, 12 | 7-day ambulatory ECG | 90 | Yes |
| Shim | 2017 | 1, 3, 6, 12 | ECG, 24-hour Holter/event recorder (symptom‑triggered) | 90 | No |
| Shin | 2021 | 1, 3, 6, 9, 12 | ECG, 24-h Holter | 90 | Yes |
| Sohns | 2023 | 3, 6, 9, 12, 24 | ILR/Implanted cardiac device monitoring | 90 | No |
| Stabile | 2006 | 1, 4, 7, 10, 13 | Periodic ECG, Transtelephonic monitor/Event monitor, Holter of unspecified duration, Echocardiogram | 30 | Yes |
| Steven | 2013 | 3, 6, 12 | ECG + 72-h Holter or 7-day event monitor | 90 | No |
| Strazdas | 2025 | 6, 12 | ECG, 24-h Holter | 60 | Yes |
| Sultan | 2025 | 3, 12 | ECG, 24-hour Holter | 90 | No |
| Sun | 2022 | 3, 6, 9, 12 | 24-hour Holter (3,6,9 months), 14-day holter (12 months) | 90 | No |
| Takahashi | 2024 | 3, 6, 12 & then 6 monthly | ECG, 24-hour Holter | 90 | No |
| Tamborero | 2010 | 1, 3, 6, 12 | ECG, 48-hour Holter | 90 | No |
| Tamborero | 2009 | 1, 4, 7, 13 | ECG, 48-hour Holter, event evaluation | 90 | No |
| Theis | 2022 | 3, 6, 9, 12 | 48-hour Holter | 90 | No |
| Theis | 2015 | 3, 6, 9, 12 | 12-lead ECG + 96-h Holter + event recorder | 90 | No |
| Turco | 2007 | 1, 4, 7, 10, 13 | Transtelephonic ECG, weekly standard ECG, ambulatory ECG | 30 | Yes |
| Ullah | 2016 | 3, 6, 12 | ECG, 7-day Holter | 90 | No |
| Ullah | 2014 | 3, 6, 12 | ECG, 7-day Holter | 90 | No |
| Wang | 2021 | 1, 2, 3, 4, 5, 6, 7, 8, 9, 10, 11, 12 | ECG, 24-hour Holter, telephone follow‑up | 90 | Yes |
| Wang | 2008 | 1, 2, 3, 6, 9, 12 | ECG, 24-hour Holter, symptom-triggered ECG, monthly phone | 30 | Yes |
| Wazni | 2020 | 1, 3, 6, 9, 12 | Periodic ECG, 24-hour Holter, Transtelephonic monitor/Event monitor | 90 | No |
| Wilber | 2010 | 1, 3, 6, 9, 12 | Transtelephonic monitor/Event monitor, 24-hour Holter | 90 | No |
| Wong | 2015 | 3, 12 | ECG, ambulatory ECG/Holter | 90 | Yes |
| Wu | 2021 | 3, 6, 12, 18, 24, 30, 36, 42, 48, 54, 60 | 7-day Holter, Transtelephonic monitor/Event monitor | 90 | No |
| Wynn | 2016 | 3,6,12 | ECG + 24-h Holter; 7–14-day monitoring if symptomatic | 90 | No |
| Xu | 2020 | 3, 6, 12, 18 | ECG (event recorder), 24-hour Holter, 7-day Holter | 90 | No |
| Xu | 2012 | 1, 3, 6 | 24-hour Holter, prior ECG, Transtelephonic monitor/Event monitor, Quality-of-life questionnaire | 0 | No |
| Yamaji | 2020 | 1, 3, 12 & then 6 monthly | ECG, telemetry ECG recorder (daily transmissions for 2 weeks), Holter as needed | 90 | No |
| Yang | 2022 | 3, 6, 12, 18 | ECG, 24-hour Holter, 7-day Holter (12 months) | 90 | No |
| Yang | 2017 | 1, 3, 6, 12 & then 6 monthly | ECG, 24-hour Holter, 7-day Holter (12 months) | 90 | No |
| Yao | 2020 | 1, 3, 6, 12, 18, 24 | ECG, 24-hour Holter | 90 | No |
| Yu | 2017 | 1, 3, 6, 12 & then 6 monthly | ECG, 24-hour Holter, event recorder | 90 | Yes |
| Zhang | 2017 | 3, 6, 12 | ECG, 24-hour Holter | NR | No |

**References**

(1-129)

1.	Ahn J, Shin DG, Han SJ, Lim HE. Does isolation of the left atrial posterior wall using cryoballoon ablation improve clinical outcomes in patients with persistent atrial fibrillation? A prospective randomized controlled trial. Europace 2022;24:1093EP–1101.

2.	Ahn J, Shin DG, Han SJ, Lim HE. Safety and efficacy of intracardiac echocardiography-guided zero-fluoroscopic cryoballoon ablation for atrial fibrillation: a prospective randomized controlled trial. Europace 2023;25.

3.	Al-Kaisey AM, Parameswaran R, Bryant C et al. Atrial Fibrillation Catheter Ablation vs Medical Therapy and Psychological Distress: A Randomized Clinical Trial. JAMA 2023;330:925EP–933.

4.	Andrade JG, Wells GA, Deyell MW et al. Cryoablation or drug therapy for initial treatment of atrial fibrillation. New England Journal of Medicine 2021;384:305EP–315.

5.	Arbelo E, Guiu E, Bisbal F et al. Benefit of left atrial roof linear ablation in paroxysmal atrial fibrillation: A prospective, randomized study. Journal of the American Heart Association 2014;3:e000877.

6.	Arentz T, Weber R, Burkle G et al. Small or large isolation areas around the pulmonary veins for the treatment of atrial fibrillation? Results from a prospective randomized study. Circulation 2007;115:3057–63.

7.	Ariyaratnam JP, Middeldorp ME, Brooks AG et al. Coronary Sinus Isolation for High-Burden Atrial Fibrillation: A Randomized Clinical Trial. JACC Clinical electrophysiology 2025;11:1–9.

8.	Aryana A, Allen SL, Pujara DK et al. Concomitant Pulmonary Vein and Posterior Wall Isolation Using Cryoballoon With Adjunct Radiofrequency in Persistent Atrial Fibrillation. JACC: Clinical Electrophysiology 2021;7:187EP–196.

9.	Baek YS, Kwon OS, Lim B et al. Clinical Outcomes of Computational Virtual Mapping-Guided Catheter Ablation in Patients With Persistent Atrial Fibrillation: A Multicenter Prospective Randomized Clinical Trial. Frontiers in Cardiovascular Medicine 2021;8:772665.

10.	Baimbetov A, Bizhanov K, Yakupova I et al. Efficacy and Safety Results of Different Ablation Technologies for Persistent Atrial Fibrillation Treatment. Heart Surgery Forum 2022;25:E594EP–E600.

11.	Bansch D, Bittkau J, Schneider R et al. Circumferential pulmonary vein isolation: wait or stop early after initial successful pulmonary vein isolation? Europace : European pacing, arrhythmias, and cardiac electrophysiology : journal of the working groups on cardiac pacing, arrhythmias, and cardiac cellular electrophysiology of the European Society of Cardiology 2013;15:183–8.

12.	Blomstrom-Lundqvist C, Gizurarson S, Schwieler J et al. Effect of Catheter Ablation vs Antiarrhythmic Medication on Quality of Life in Patients with Atrial Fibrillation: The CAPTAF Randomized Clinical Trial. JAMA - Journal of the American Medical Association 2019;321:1059EP–1068.

13.	Brachmann J, Vogtmann T, Hummel J et al. Prospective Randomized Trial of Rotor Ablation vs Conventional Ablation for Persistent Atrial Fibrillation: REAFFIRM Trial. Journal of the American Heart Association 2025;14:e022346.

14.	Buist TJ, Adiyaman A, Smit JJJ, Ramdat Misier AR, Elvan A. Arrhythmia-free survival and pulmonary vein reconnection patterns after second-generation cryoballoon and contact-force radiofrequency pulmonary vein isolation. Clinical research in cardiology : official journal of the German Cardiac Society 2018;107:498–506.

15.	Cao Y, Du W, Fei Y et al. Superwide Left Atrial Circumferential Ablation for Persistent Atrial Fibrillation: A Randomized Controlled Trial. Journal of Cardiovascular Electrophysiology 2025;36:1530EP–1537.

16.	Caponi D, Corleto A, Scaglione M et al. Ablation of atrial fibrillation: Does the addition of three-dimensional magnetic resonance imaging of the left atrium to electroanatomic mapping improve the clinical outcome? Europace 2010;12:1098EP–1104.

17.	Castro-Urda V, Segura-Dominguez M, Jimenez-Sanchez D et al. Superior Vena Cava Isolation with Cryoballoon in AF Ablation: Randomized CAVAC AF Trial. Circulation: Arrhythmia and Electrophysiology 2025;18:e012917.

18.	Chen H, Li C, Han B et al. Circumferential Pulmonary Vein Isolation with vs Without Additional Low-Voltage-Area Ablation in Older Patients with Paroxysmal Atrial Fibrillation: A Randomized Clinical Trial. JAMA Cardiology 2023;8:765EP–772.

19.	Choi Y, Lim B, Yang SY et al. Clinical Usefulness of Virtual Ablation Guided Catheter Ablation of Atrial Fibrillation Targeting Restitution Parameter-Guided Catheter Ablation: CUVIA-REGAB Prospective Randomized Study. Korean Circulation Journal 2022;52:e75.

20.	Chun JKR, Bordignon S, Last J et al. Cryoballoon Versus Laserballoon: Insights From the First Prospective Randomized Balloon Trial in Catheter Ablation of Atrial Fibrillation. Circulation: Arrhythmia and Electrophysiology 2021;14:E009294.

21.	Corrado A, Bonso A, Madalosso M et al. Impact of systematic isolation of superior vena cava in addition to pulmonary vein antrum isolation on the outcome of paroxysmal, persistent, and permanent atrial fibrillation ablation: results from a randomized study. Journal of cardiovascular electrophysiology 2010;21:1–5.

22.	Darkner S, Chen X, Hansen J et al. Recurrence of arrhythmia following short-term oral AMIOdarone after CATheter ablation for atrial fibrillation: A double-blind, randomized, placebo-controlled study (AMIO-CAT trial). European Heart Journal 2014;35:3356EP–3364.

23.	Deisenhofer I, Albenque JP, Busch S et al. Artificial intelligence for individualized treatment of persistent atrial fibrillation: a randomized controlled trial. Nature Medicine 2025;31:1286EP–1293.

24.	Di Biase L, Mohanty P, Mohanty S et al. Ablation Versus Amiodarone for Treatment of Persistent Atrial Fibrillation in Patients With Congestive Heart Failure and an Implanted Device: Results From the AATAC Multicenter Randomized Trial. Circulation 2016;133:1637EP–1644.

25.	Ding J, Cheng A, Li P et al. Cryoballoon catheter ablation or drug therapy to delay progression of atrial fibrillation: A single-center randomized trial. Frontiers in Cardiovascular Medicine 2022;9:1003305.

26.	Dixit S, Gerstenfeld EP, Ratcliffe SJ et al. Single procedure efficacy of isolating all versus arrhythmogenic pulmonary veins on long-term control of atrial fibrillation: A prospective randomized study. Heart Rhythm 2008;5:174EP–181.

27.	Dong JZ, Sang CH, Yu RH et al. Prospective randomized comparison between a fixed '2C3L' approach vs. stepwise approach for catheter ablation of persistent atrial fibrillation. Europace 2015;17:1798EP–1806.

28.	Dukkipati SR, Cuoco F, Kutinsky I et al. Pulmonary Vein Isolation Using the Visually Guided Laser Balloon A Prospective, Multicenter, and Randomized Comparison to Standard Radiofrequency Ablation. Journal of the American College of Cardiology 2015;66:1350EP–1360.

29.	Dulai R, Sulke N, Freemantle N et al. Pulmonary Vein Isolation vs Sham Intervention in Symptomatic Atrial Fibrillation: The SHAM-PVI Randomized Clinical Trial. JAMA 2024;332:1165EP–1173.

30.	Duytschaever M, Demolder A, Phlips T et al. P ulm O nary vein isolation W ith vs. without continued antiarrhythmic D rug tr e atment in subjects with R ecurrent A trial F ibrillation (POWDER AF): Results from a multicentre randomized trial. European Heart Journal 2018;39:1429EP–1437.

31.	Ejima K, Kato K, Okada A et al. Comparison Between Contact Force Monitoring and Unipolar Signal Modification as a Guide for Catheter Ablation of Atrial Fibrillation: Prospective Multi-Center Randomized Study. Circulation: Arrhythmia and Electrophysiology 2019;12:007311.

32.	Estner HL, Hessling G, Biegler R et al. Complex fractionated atrial electrogram or linear ablation in patients with persistent atrial fibrillation-a prospective randomized study. PACE - Pacing and Clinical Electrophysiology 2011;34:939EP–948.

33.	Falasconi G, Penela D, Soto-Iglesias D et al. Personalized pulmonary vein isolation guided by left atrial wall thickness for persistent atrial fibrillation ablation: the PeAF-by-LAWT randomized trial. Europace : European pacing, arrhythmias, and cardiac electrophysiology : journal of the working groups on cardiac pacing, arrhythmias, and cardiac cellular electrophysiology of the European Society of Cardiology 2025.

34.	Fassini G, Riva S, Chiodelli R et al. Left mitral isthmus ablation associated with PV isolation: Long-term results of a prospective randomized study. Journal of Cardiovascular Electrophysiology 2005;16:1150EP–1156.

35.	Faustino M, Pizzi C, Agricola T et al. Stepwise ablation approach versus pulmonary vein isolation in patients with paroxysmal atrial fibrillation: Randomized controlled trial. Heart Rhythm 2015;12:1907EP–1915.

36.	Ferrero-De-Loma-Osorio A, Garcia-Fernandez A, Castillo-Castillo J et al. Time-to-Effect-Based Dosing Strategy for Cryoballoon Ablation in Patients with Paroxysmal Atrial Fibrillation: Results of the plusONE Multicenter Randomized Controlled Noninferiority Trial. Circulation: Arrhythmia and Electrophysiology 2017;10:e005318.

37.	Fiala M, Chovancik J, Nevralova R et al. Pulmonary vein isolation using segmental versus electroanatomical circumferential ablation for paroxysmal atrial fibrillation: Over 3-year results of a prospective randomized study. Journal of Interventional Cardiac Electrophysiology 2008;22:13EP–21.

38.	Fichtner S, Hessling G, Ammar S et al. A prospective randomized study comparing isolation of the arrhythmogenic vein versus all veins in paroxysmal atrial fibrillation. Clinical Cardiology 2013;36:422EP–426.

39.	Fink T, Schluter M, Heeger CH et al. Stand-Alone Pulmonary Vein Isolation Versus Pulmonary Vein Isolation with Additional Substrate Modification as Index Ablation Procedures in Patients with Persistent and Long-Standing Persistent Atrial Fibrillation: The Randomized Alster-Lost-AF Trial (Abl. Circulation: Arrhythmia and Electrophysiology 2017;10:e005114.

40.	Gaita F, Caponi D, Scaglione M et al. Long-term clinical results of 2 different ablation strategies in patients with paroxysmal and persistent atrial fibrillation. Circulation Arrhythmia and electrophysiology 2008;1:269EP–275.

41.	Gal P, Aarntzen AESM, Smit JJJ et al. Conventional radiofrequency catheter ablation compared to multi-electrode ablation for atrial fibrillation. International journal of cardiology 2014;176:891–5.

42.	Gallagher MM, Yi G, Gonna H et al. Multi-catheter cryotherapy compared with radiofrequency ablation in long-standing persistent atrial fibrillation: A randomized clinical trial. Europace 2021;23:370EP–379.

43.	Gula LJ, Leong-Sit P, Manlucu J et al. Pulmonary Vein Isolation With Incomplete Antral Ablation Lines: Is More Ablation Necessary? Results of a Randomized Trial. Journal of cardiovascular electrophysiology 2016;27:298–302.

44.	Inoue K, Hikoso S, Masuda M et al. Pulmonary vein isolation alone vs. more extensive ablation with defragmentation and linear ablation of persistent atrial fibrillation: The EARNEST-PVI trial. Europace 2021;23:565EP–574.

45.	Jais P, Cauchemez B, Macle L et al. Catheter ablation versus antiarrhythmic drugs for atrial fibrillation: The A4 study. Circulation 2008;118:2498EP–2505.

46.	Jiang R, Chen M, Fan J et al. Efficacy of ablation index-guided pulmonary vein isolation in patients with paroxysmal atrial fibrillation. PACE - Pacing and Clinical Electrophysiology 2022;45:1186EP–1193.

47.	Kaiser B, Huber C, Pirozzolo G, Maier P, Bekeredjian R, Theis C. Persistent atrial fibrillation without the evidence of low-voltage areas: a prospective randomized trial. Journal of Interventional Cardiac Electrophysiology 2024;67:83EP–90.

48.	Kalman JM, Al-Kaisey AM, Parameswaran R et al. Impact of early vs. delayed atrial fibrillation catheter ablation on atrial arrhythmia recurrences. European heart journal 2023;44:2447–2454.

49.	Kang KW, Pak HN, Park J et al. Additional linear ablation from the superior vena cava to right atrial septum after pulmonary vein isolation improves the clinical outcome in patients with paroxysmal atrial fibrillation: Prospective randomized study. Europace 2014;16:1738EP–1745.

50.	Karch MR, Zrenner B, Deisenhofer I et al. Freedom from atrial tachyarrhythmias after catheter ablation of atrial fibrillation: A randomized comparison between 2 current ablation strategies. Circulation 2005;111:2875EP–2880.

51.	Kim D, Kwon O-S, Hwang T et al. Using computed tomography atrial myocardial thickness maps in cryoballoon pulmonary vein isolation: the UTMOST AF II randomized clinical trial. Europace : European pacing, arrhythmias, and cardiac electrophysiology : journal of the working groups on cardiac pacing, arrhythmias, and cardiac cellular electrophysiology of the European Society of Cardiology 2024;26.

52.	Kim D, Yu HT, Shim J et al. Cryoballoon Pulmonary Vein Isolation with Versus Without Additional Right Atrial Linear Ablation for Persistent Atrial Fibrillation: The CRALAL Randomized Clinical Trial. Circulation: Arrhythmia and Electrophysiology 2025;18:e013408.

53.	Kin YH, Lim HE, Pak HN et al. Role of residual potentials inside circumferential pulmonary veins ablation lines in the recurrence of paroxysmal atrial fibrillation. Journal of Cardiovascular Electrophysiology 2010;21:959EP–965.

54.	Kircher S, Arya A, Altmann D et al. Individually tailored vs. standardized substrate modification during radiofrequency catheter ablation for atrial fibrillation: A randomized study. Europace 2018;20:1766EP–1775.

55.	Kistler PM, Chieng D, Sugumar H et al. Effect of Catheter Ablation Using Pulmonary Vein Isolation with vs Without Posterior Left Atrial Wall Isolation on Atrial Arrhythmia Recurrence in Patients with Persistent Atrial Fibrillation: The CAPLA Randomized Clinical Trial. JAMA 2023;329:127EP–135.

56.	Kobori A, Shizuta S, Inoue K et al. Adenosine triphosphate-guided pulmonary vein isolation for atrial fibrillation: The UNmasking dormant electrical reconduction by adenosine triphosphate (UNDER-ATP) trial. European Heart Journal 2015;36:3276EP–3287.

57.	Kuck KH, Brugada J, Furnkranz A et al. Cryoballoon or radiofrequency ablation for paroxysmal atrial fibrillation. New England Journal of Medicine 2016;374:2235EP–2245.

58.	Kuck KH, Lebedev DS, Mikhaylov EN et al. Catheter ablation or medical therapy to delay progression of atrial fibrillation: The randomized controlled atrial fibrillation progression trial (ATTEST). Europace 2021;23:362EP–369.

59.	Kuck KH, Merkely B, Zahn R et al. Catheter Ablation Versus Best Medical Therapy in Patients With Persistent Atrial Fibrillation and Congestive Heart Failure: The Randomized AMICA Trial. Circulation: Arrhythmia and Electrophysiology 2019;12:e007731.

60.	Kuniss M, Pavlovic N, Velagic V et al. Cryoballoon ablation vs. antiarrhythmic drugs: First-line therapy for patients with paroxysmal atrial fibrillation. Europace 2021;23:1033EP–1041.

61.	Lee JM, Shim J, Park J et al. The Electrical Isolation of the Left Atrial Posterior Wall in Catheter Ablation of Persistent Atrial Fibrillation. JACC: Clinical Electrophysiology 2019;5:1253EP–1261.

62.	Lee KN, Choi JI, Kim YG et al. Comparison between linear and focal ablation of complex fractionated atrial electrograms in patients with non-paroxysmal atrial fibrillation: A prospective randomized trial. Europace 2019;21:598EP–606.

63.	Lee KN, Roh SY, Baek YS et al. Long-Term Clinical Comparison of Procedural End Points after Pulmonary Vein Isolation in Paroxysmal Atrial Fibrillation: Elimination of Nonpulmonary Vein Triggers Versus Noninducibility. Circulation: Arrhythmia and Electrophysiology 2018;11:e005019.

64.	Lin CY, Lin YJ, Higa S et al. Catheter Ablation With Morphologic Repetitiveness Mapping for Persistent Atrial Fibrillation. JAMA Network Open 2023;6:E2344535.

65.	Lin R, Zeng C, Xu K, Wu S, Qin M, Liu X. Dispersion-guided ablation in conjunction with circumferential pulmonary vein isolation is superior to stepwise ablation approach for persistent atrial fibrillation. International Journal of Cardiology 2019;278:97EP–103.

66.	Lin Y-J, Chang S-L, Lo L-W et al. A prospective, randomized comparison of modified pulmonary vein isolation versus conventional pulmonary vein isolation in patients with paroxysmal atrial fibrillation. Journal of cardiovascular electrophysiology 2012;23:1155–62.

67.	Liu X, Long D, Dong J et al. Is circumferential pulmonary vein isolation preferable to stepwise segmental pulmonary vein isolation for patients with paroxysmal atrial fibrillation? Circulation journal : official journal of the Japanese Circulation Society 2006;70:1392–7.

68.	Luik A, Radzewitz A, Kieser M et al. Cryoballoon Versus Open Irrigated Radiofrequency Ablation in Patients With Paroxysmal Atrial Fibrillation: The Prospective, Randomized, Controlled, Noninferiority FreezeAF Study. Circulation 2015;132:1311–9.

69.	Malmborg H, Lonnerholm S, Blomstrom P, Blomstrom-Lundqvist C. Ablation of atrial fibrillation with cryoballoon or duty-cycled radiofrequency pulmonary vein ablation catheter: A randomized controlled study comparing the clinical outcome and safety; The AF-COR study. Europace 2013;15:1567EP–1573.

70.	Mamchur S, Chichkova T, Khomenko E, Kokov A. Pulmonary Veins Morphometric Characteristics and Spatial Orientation Influence on Its Cryoballoon Isolation Results. Diagnostics 2022;12:1322.

71.	Marrouche NF, Brachmann J, Andresen D et al. Catheter ablation for atrial fibrillation with heart failure. New England Journal of Medicine 2018;378:417EP–427.

72.	Marrouche NF, Wazni O, McGann C et al. Effect of MRI-Guided Fibrosis Ablation vs Conventional Catheter Ablation on Atrial Arrhythmia Recurrence in Patients With Persistent Atrial Fibrillation: The DECAAF II Randomized Clinical Trial. JAMA 2022;327:2296EP–2305.

73.	Masuda M, Sunaga A, Tanaka N et al. Low-voltage-area ablation for persistent atrial fibrillation: a randomized controlled trial. Nature Medicine 2025;31:1661EP–1667.

74.	McLellan AJA, Ling LH, Azzopardi S et al. A minimal or maximal ablation strategy to achieve pulmonary vein isolation for paroxysmal atrial fibrillation: A prospective multi-centre randomized controlled trial (the minimax study). Heart Rhythm 2015;12:S263.

75.	Mililis P, Kariki O, Saplaouras A et al. Radiofrequency versus cryoballoon catheter ablation in patients with persistent atrial fibrillation: A randomized trial. Journal of Cardiovascular Electrophysiology 2023;34:1523EP–1528.

76.	Miyamoto K, Kanaoka K, Yodogawa K et al. Cryoballoon vs radiofrequency ablation in persistent atrial fibrillation: the CRRF-PeAF trial. European heart journal 2025.

77.	Mohanty S, Mohanty P, Di Biase L et al. Results from a single-blind, randomized study comparing the impact of different ablation approaches on long-term procedure outcome in coexistent atrial fibrillation and flutter (APPROVAL). Circulation 2013;127:1853–60.

78.	Mont L, Bisbal F, Hernandez-Madrid A et al. Catheter ablation vs. antiarrhythmic drug treatment of persistent atrial fibrillation: A multicentre, randomized, controlled trial (SARA study). European Heart Journal 2014;35:501EP–507.

79.	Morillo CA, Verma A, Connolly SJ et al. Radiofrequency ablation vs antiarrhythmic drugs as first-line treatment of paroxysmal atrial fibrillation (RAAFT-2) a randomized trial. JAMA 2014;311:692EP–699.

80.	Nielsen JC, Johannessen A, Raatikainen P et al. Radiofrequency ablation as initial therapy in paroxysmal atrial fibrillation. New England Journal of Medicine 2012;367:1587EP–1595.

81.	Nilsson B, Chen X, Pehrson S, Kober L, Hilden J, Svendsen JH. Recurrence of pulmonary vein conduction and atrial fibrillation after pulmonary vein isolation for atrial fibrillation: A randomized trial of the ostial versus the extraostial ablation strategy. American Heart Journal 2006;152:537.e1EP–537.e8.

82.	Oral H, Pappone C, Chugh A et al. Circumferential pulmonary-vein ablation for chronic atrial fibrillation. New England Journal of Medicine 2006;354:934EP–941.

83.	Osmancik P, Roubicek T, Havranek S et al. Catheter Ablation vs Lifestyle Modification With Antiarrhythmic Drugs to Treat Atrial Fibrillation: PRAGUE-25 Trial. Journal of the American College of Cardiology 2025;86:18EP–28.

84.	Packer DL, Kowal RC, Wheelan KR et al. Cryoballoon ablation of pulmonary veins for paroxysmal atrial fibrillation: First results of the North American arctic front (STOP AF) pivotal trial. Journal of the American College of Cardiology 2013;61:1713EP–1723.

85.	Packer DL, Mark DB, Robb RA et al. Catheter ablation vs. antiarrhythmic drug therapy for atrial fibrillation: The results of the cabana multicenter international randomized clinical trial. Heart Rhythm 2018;15:940EP–941.

86.	Pak HN, Park J, Park JW et al. Electrical Posterior Box Isolation in Persistent Atrial Fibrillation Changed to Paroxysmal Atrial Fibrillation: A Multicenter, Prospective, Randomized Study. Circulation: Arrhythmia and Electrophysiology 2020;13:E008531.

87.	Pak HN, Park JW, Yang SY et al. Cryoballoon Versus High-Power, Short-Duration Radiofrequency Ablation for Pulmonary Vein Isolation in Patients With Paroxysmal Atrial Fibrillation: A Single-Center, Prospective, Randomized Study. Circulation: Arrhythmia and Electrophysiology 2021;14:E010040.

88.	Pappone C, Augello G, Sala S et al. A randomized trial of circumferential pulmonary vein ablation versus antiarrhythmic drug therapy in paroxysmal atrial fibrillation: the APAF Study. Journal of the American College of Cardiology 2006;48:2340–7.

89.	Parkash R, Wells GA, Rouleau J et al. Randomized Ablation-Based Rhythm-Control Versus Rate-Control Trial in Patients with Heart Failure and Atrial Fibrillation: Results from the RAFT-AF trial. Circulation 2022;145:1693EP–1704.

90.	Pavlovic N, Kuniss M, Velagic V et al. Impact of initial rhythm control with cryoballoon ablation versus drug therapy on atrial fibrillation recurrence and quality of life: Results from the Cryo-FIRST study. Europace 2021;23:iii236EP–iii267.

91.	Reddy VY, Gerstenfeld EP, Natale A et al. Pulsed Field or Conventional Thermal Ablation for Paroxysmal Atrial Fibrillation. New England Journal of Medicine 2023;389:1660EP–1671.

92.	Reynolds MR, Walczak J, White SA, Cohen DJ, Wilber DJ. Improvements in symptoms and quality of life in patients with paroxysmal atrial fibrillation treated with radiofrequency catheter ablation versus antiarrhythmic drugs. Circulation: Cardiovascular Quality and Outcomes 2010;3:615EP–623.

93.	Rillig A, Schmidt B, Di Biase L et al. Manual Versus Robotic Catheter Ablation for the Treatment of Atrial Fibrillation: The Man and Machine Trial. JACC: Clinical Electrophysiology 2017;3:875EP–883.

94.	Sang C, Liu Q, Lai Y et al. Pulmonary Vein Isolation With Optimized Linear Ablation vs Pulmonary Vein Isolation Alone for Persistent AF: The PROMPT-AF Randomized Clinical Trial. JAMA 2025;333:381EP–389.

95.	Schmidt B, Neuzil P, Luik A et al. Laser Balloon or Wide-Area Circumferential Irrigated Radiofrequency Ablation for Persistent Atrial Fibrillation: A Multicenter Prospective Randomized Study. Circulation Arrhythmia and electrophysiology 2017;10.

96.	Sheikh I, Krum D, Cooley R et al. Pulmonary vein isolation and linear lesions in atrial fibrillation ablation. Journal of interventional cardiac electrophysiology : an international journal of arrhythmias and pacing 2006;17:103–9.

97.	Shen W, Zhang Y, Qian W et al. Pulmonary Vein Isolation With or Without Empiric Superior Vena Cava Isolation in Patients Undergoing Ablation for Paroxysmal Atrial Fibrillation: The Randomized ESVCI-AF Trial. Europace : European pacing, arrhythmias, and cardiac electrophysiology : journal of the working groups on cardiac pacing, arrhythmias, and cardiac cellular electrophysiology of the European Society of Cardiology 2025.

98.	Shi LB, Rossvoll O, Tande P, Schuster P, Solheim E, Chen J. Cryoballoon vs. radiofrequency catheter ablation: insights from NOrwegian randomized study of PERSistent Atrial Fibrillation (NO-PERSAF study). Europace 2022;24:226EP–233.

99.	Shim J, Hwang M, Song JS et al. Virtual in-silico modeling guided catheter ablation predicts effective linear ablation lesion set for longstanding persistent atrial fibrillation: Multicenter prospective randomized study. Frontiers in Physiology 2017;8:792.

100.	Shin DG, Roh SY, Ahn J et al. Does merged three-dimensional mapping improve contact force and long-term procedure outcome in atrial fibrillation ablation? (MICRO-AF study): a prospective randomized controlled study. International Journal of Cardiovascular Imaging 2021;37:3431EP–3438.

101.	Sohns C, Fox H, Marrouche NF et al. Catheter Ablation in End-Stage Heart Failure with Atrial Fibrillation. New England Journal of Medicine 2023;389:1380EP–1389.

102.	Stabile G, Bertaglia E, Senatore G et al. Catheter ablation treatment in patients with drug-refractory atrial fibrillation: A prospective, multi-centre, randomized, controlled study (Catheter Ablation for the Cure of Atrial Fibrillation Study). European Heart Journal 2006;27:216EP–221.

103.	Steven D, Sultan A, Reddy V et al. Benefit of pulmonary vein isolation guided by loss of pace capture on the ablation line: Results from a prospective 2-center randomized trial. Journal of the American College of Cardiology 2013;62:44EP–50.

104.	Strazdas A, Tijskens M, Abugattas JP, Wolf M, Schwagten B, De Greef Y. Adding Non-Occlusive Cryoballoon Ablation in Case of Ostial-Level Pulmonary Vein Isolation or Carina Sparing Does Not Improve 1-Year Outcome After Cryoballoon Ablation (the NOCA-AF Study). Journal of cardiovascular electrophysiology 2025;36:2330–2340.

105.	Sultan A, Kreutzer S, Wormann J et al. HIgh Power short duration radiofrequency ablation or cryoballoon ablation for paroxysmal Atrial Fibrillation (HIPAF trial). Europace 2025;27:euaf066.

106.	Sun J, Chen M, Wang Q et al. Adding six short lines on pulmonary vein isolation circumferences reduces recurrence of paroxysmal atrial fibrillation: Results from a multicenter, single-blind, randomized trial. Heart rhythm 2022;19:344–351.

107.	Takahashi Y, Kobori A, Hiroshima K et al. Mapping-Guided Ablation for Persistent Atrial Fibrillation (MAP-AF): A Multicenter, Single-Blind, Randomized Controlled Trial. Circulation: Arrhythmia and Electrophysiology 2024;17:e012829.

108.	Tamborero D, Mont L, Berruezo A et al. Circumferential pulmonary vein ablation: Does use of a circular mapping catheter improve results? A prospective randomized study. Heart Rhythm 2010;7:612EP–618.

109.	Tamborero D, Mont L, Berruezo A et al. Left atrial posterior wall isolation does not improve the outcome of circumferential pulmonary vein ablation for atrial fibrillation: A prospective randomized study. Circulation: Arrhythmia and Electrophysiology 2009;2:35EP–40.

110.	Theis C, Huber C, Kaiser B, Kaesemann P, Pirozzolo G, Bekeredjian R. Pulmonary vein isolation using Cryo Balloon ablation versus RF ablation using ablation index following the CLOSE protocol: A Prospective Randomized Trial. Europace 2021;23:iii261.

111.	Theis C, Konrad T, Mollnau H et al. Arrhythmia Termination Versus Elimination of Dormant Pulmonary Vein Conduction as a Procedural End Point of Catheter Ablation for Paroxysmal Atrial Fibrillation. Circulation: Arrhythmia and Electrophysiology 2015;8:1080EP–1087.

112.	Turco P, De Simone A, La Rocca V et al. Antiarrhythmic drug therapy after radiofrequency catheter ablation in patients with atrial fibrillation. Pacing and clinical electrophysiology : PACE 2007;30 Suppl 1:S112–5.

113.	Ullah W, McLean A, Hunter RJ et al. Randomized trial comparing robotic to manual ablation for atrial fibrillation. Heart Rhythm 2014;11:1862EP–1869.

114.	Ullah W, McLean A, Tayebjee MH et al. Randomized trial comparing pulmonary vein isolation using the SmartTouch catheter with or without real-time contact force data. Heart rhythm 2016;13:1761–7.

115.	Wang W, Chen Q, Zhang F et al. Radiofrequency catheter ablation combined with spironolactone in the treatment of atrial fibrillation: A single-center randomized controlled study. Clinical Cardiology 2021;44:1120EP–1127.

116.	Wang XH, Liu X, Sun YM, Shi HF, Zhou L, Gu JN. Pulmonary vein isolation combined with superior vena cava isolation for atrial fibrillation ablation: A prospective randomized study. Europace 2008;10:600EP–605.

117.	Wazni OM, Dandamudi G, Sood N et al. Cryoballoon ablation as initial therapy for atrial fibrillation. New England Journal of Medicine 2021;384:316EP–324.

118.	Wilber DJ, Pappone C, Neuzil P et al. Comparison of antiarrhythmic drug therapy and radiofrequency catheter ablation in patients with paroxysmal atrial fibrillation: A randomized controlled trial. JAMA 2010;303:333EP–340.

119.	Wong KCK, Paisey JR, Sopher M et al. No Benefit of Complex Fractionated Atrial Electrogram Ablation in Addition to Circumferential Pulmonary Vein Ablation and Linear Ablation: Benefit of Complex Ablation Study. Circulation: Arrhythmia and Electrophysiology 2015;8:1316EP–1324.

120.	Wu G, Huang H, Cai L et al. Long-term observation of catheter ablation vs. pharmacotherapy in the management of persistent and long-standing persistent atrial fibrillation (CAPA study). Europace 2021;23:731EP–739.

121.	Wynn GJ, Panikker S, Morgan M et al. Biatrial linear ablation in sustained nonpermanent AF: Results of the substrate modification with ablation and antiarrhythmic drugs in nonpermanent atrial fibrillation (SMAN-PAF) trial. Heart Rhythm 2016;13:399EP–406.

122.	Xu C, Zhang F, Chen W et al. Adjunctive Right Atrial Ablation for Persistent Atrial Fibrillation With Right Atrial Enlargement: A Pilot Study. Canadian Journal of Cardiology 2024;40:564EP–572.

123.	Xu Q, Ju W, Xiao F et al. Circumferential pulmonary vein antrum ablation for the treatment of paroxysmal atrial fibrillation: A randomized controlled trial. Pacing and clinical electrophysiology : PACE 2020;43:280–288.

124.	Yamaji H, Higashiya S, Murakami T et al. Efficacy of an Adjunctive Electrophysiological Test-Guided Left Atrial Posterior Wall Isolation in Persistent Atrial Fibrillation Without a Left Atrial Low-Voltage Area. Circulation: Arrhythmia and Electrophysiology 2020;13:E008191.

125.	Yang B, Jiang C, Lin Y et al. STABLE-SR (Electrophysiological substrate ablation in the left atrium during sinus rhythm) for the treatment of nonparoxysmal atrial fibrillation: A prospective, multicenter randomized clinical trial. Circulation: Arrhythmia and Electrophysiology 2017;10:e005405.

126.	Yang G, Zheng L, Jiang C et al. Circumferential Pulmonary Vein Isolation Plus Low-Voltage Area Modification in Persistent Atrial Fibrillation: The STABLE-SR-II Trial. JACC Clinical electrophysiology 2022;8:882–891.

127.	Yao Y, Hu F, Du Z et al. The value of extensive catheter linear ablation on persistent atrial fibrillation (the CLEAR-AF Study). International Journal of Cardiology 2020;316:125EP–129.

128.	Yu HT, Shim J, Park J et al. Pulmonary Vein Isolation Alone Versus Additional Linear Ablation in Patients with Persistent Atrial Fibrillation Converted to Paroxysmal Type with Antiarrhythmic Drug Therapy: A Multicenter, Prospective, Randomized Study. Circulation: Arrhythmia and Electrophysiology 2017;10:e004915.

129.	Zhang JQ, Yu RH, Liang JB et al. Reconstruction left atrium and isolation pulmonary veins of paroxysmal atrial fibrillation using single contact force catheter with zero x-ray exposure: A CONSORT Study. Medicine 2017;96:e7726.
